# RIPK3/Fas-Associated Death Domain Axis Regulates Pulmonary Immunopathology to Cryptococcal Infection Independent of Necroptosis

**DOI:** 10.3389/fimmu.2017.01055

**Published:** 2017-09-01

**Authors:** Zhenzong Fa, Qun Xie, Wei Fang, Haibing Zhang, Haiwei Zhang, Jintao Xu, Weihua Pan, Jinhua Xu, Michal A. Olszewski, Xiaoming Deng, Wanqing Liao

**Affiliations:** ^1^PLA Key Laboratory of Mycosis, Department of Dermatology and Venereology, Changzheng Hospital, Shanghai, China; ^2^Shanghai Key Laboratory of Molecular Medical Mycology, Shanghai Institute of Medical Mycology, Second Military Medical University, Shanghai, China; ^3^Division of Pulmonary and Critical Care Medicine, Department of Internal Medicine, University of Michigan Health System, Ann Arbor, MI, United States; ^4^Department of Anesthesiology and Intensive Care, Changhai Hospital, Second Military Medical University, Shanghai, China; ^5^Key Laboratory of Nutrition and Metabolism, Institute for Nutritional Sciences, Shanghai Institutes for Biological Sciences, Chinese Academy of Sciences, Shanghai, China; ^6^Department of Dermatology, Huashan Hospital, Fudan University, Shanghai, China

**Keywords:** *Cryptococcus neoformans*, immune responses, inflammation, Fas-associated death domain, receptor-interacting serine/threonine kinase 3

## Abstract

Fas-associated death domain (FADD) and receptor interacting protein kinase 3 (RIPK3) are multifunctional regulators of cell death and immune response. Using a mouse model of cryptococcal infection, the roles of FADD and RIPK3 in anti-cryptococcal defense were investigated. Deletion of RIPK3 alone led to increased inflammatory cytokine production in the *Cryptococcus neoformans*-infected lungs, but in combination with FADD deletion, it led to a robust Th1-biased response with M1-biased macrophage activation. Rather than being protective, these responses led to paradoxical *C. neoformans* expansion and rapid clinical deterioration in *Ripk3*^−/−^ and *Ripk3*^−/−^*Fadd*^−/−^ mice. The increased mortality of *Ripk3*^−/−^ and even more accelerated mortality in *Ripk3*^−/−^*Fadd*^−/−^ mice was attributed to profound pulmonary damage due to neutrophil-dominant infiltration with prominent upregulation of pro-inflammatory cytokines. This phenomenon was partially associated with selective alterations in the apoptotic frequency of some leukocyte subsets, such as eosinophils and neutrophils, in infected *Ripk3*^−/−^*Fadd*^−/−^ mice. In conclusion, our study shows that RIPK3 in concert with FADD serve as physiological “brakes,” preventing the development of excessive inflammation and Th1 bias, which in turn contributes to pulmonary damage and defective fungal clearance. This novel link between the protective effect of FADD and RIPK3 in antifungal defense and sustenance of immune homeostasis may be important for the development of novel immunomodulatory therapies against invasive fungal infections.

## Introduction

Invasive fungal infections have become an increasingly significant challenge to public health due to the ever-increasing population of immunosuppressed patients, associated with aging of the global population, immunosuppressive infections such as HIV, and the growing use of immunosuppressive therapies. Among the major fungal pathogens, *Cryptococcus neoformans* causes life-threatening invasive infections in both immunocompromised and immunocompetent hosts (1, 2). As supported by evidence from both the clinic and animal infection models, an insufficiency of Th1 and Th17 responses and subsequent classical activation of macrophages are major triggers of cryptococcal infection. However, emerging evidence supports the view that the excessive inflammation and pathology is frequently derived by a Th1 response, which is often initiated during highly active antiretroviral therapy (HAART) in HIV^+^ patients with cryptococcosis. This paradoxical response, known as immune reconstitution inflammatory syndrome (IRIS), contributes to worsening symptoms and patient mortality despite ongoing antifungal and HAART treatments (3). This unique clinical problem underscores the importance of immunoregulatory processes during opportunistic fungal infections of which many aspects remain to be elucidated.

Fas-associated death domain protein (FADD) is known as a critical mediator of death receptor-triggered extrinsic apoptosis, which plays a role in removing “no longer needed” inflammatory cells, thereby serving as a crucial immune-regulatory pathway at the site of infection, preventing excessive inflammation (4, 5). Besides its role in apoptosis, FADD also has been shown to function in regulating cell cycle progression (6, 7), cytokine signaling (8, 9), and T-cell activation (10), which are all involved in regulation of immune responses. Targeted deletion of FADD in mice causes embryonic lethality due to spontaneous activation of another programmed cell death (PCD) pathway, necroptosis (11). However, co-deletion of receptor interacting protein kinase 3 (RIPK3), which is an essential serine/threonine kinase for necroptosis, rescues these mice (12). In addition to necroptosis, RIPK3 has also been reported as an important inflammatory signal adaptor because it functions in NF-κB activation, inflammasome activation, and cytokine signaling (13–15) and participates in the pathogenesis of several inflammatory diseases. However, it remains unknown whether these molecules play important roles during immune responses to fungal infections.

Here, we explored the roles of FADD and RIPK3 in a mouse model of cryptococcal infection and identified previously unknown, critical contributions of these molecules in pulmonary immune responses to cryptococcal infection. Deletion of FADD and RIPK3 induced robust Th1 responses, which, paradoxically, led to *C. neoformans* expansion and increased mortality in the infected mice. These effects were attributed to an excessive accumulation of neutrophils, over exuberant inflammatory cytokine production, and development of severe lung pathology. Collectively, these findings establish a novel link between these PCD components and immune response to cryptococcal challenge, demonstrating the crucial importance of FADD and RIPK3 in maintaining immune homeostasis during invasive fungal infection.

## Materials and Methods

### Mice

Female wild-type (WT) C57BL/6 mice were housed in a specific pathogen-free facility. *Ripk3*^−/−^ mice have been previously described (16). *Fadd*^+/−^ mice were generated using the CRISPR–Cas9 mutation system (Shanghai Bioray Laboratory, Inc.). A 100-bp deletion was introduced into exon 1 of the *Fadd* gene (Figure S1 in Supplementary Material). Because ablation of *Fadd* in mice causes embryonic death, we crossed the *Ripk3*^−/−^ mice with *Fadd*^+/−^ mice to obtain *Ripk3*^−/−^*Fadd*^−/−^ mice. All mice genotypes were confirmed by PCR (Figure S1 in Supplementary Material). Mice were 8–10 weeks old at the time of infection and were humanely euthanized by CO_2_ inhalation at the time of data collection. Animal experiments were conducted in accordance with the National Institutes of Health Guide for the Care and Use of Laboratory Animals with the approval of the Scientific Investigation Board of Second Military Medical University.

### *Cryptococcus* *neoformans*

Encapsulated *C. neoformans* strain H99 (serotype A) was recovered from 10% glycerol-frozen stocks stored at −80°C. The strains were cultured on yeast extract-peptone-dextrose agar plates at 30°C. Liquid cultures were grown in Sabouraud dextrose broth at 30°C for 20–24 h in a shaking incubator at 180 rpm. Fungal cells were centrifuged at 2,000 × *g* for 3 min, washed three times, and resuspended in sterile PBS.

### Inoculation

Mice were anesthetized by intraperitoneal injection of ketamine (100 mg/kg, Sigma, St. Louis, MO, USA) and secured onto a clean foam board (17). Next, 50 μl [10^5^ colony-forming units (CFU)] of the washed yeast (2 × 10^6^ yeast cells/ml in sterile PBS) were used for intranasal infection as previously described (18). After inoculation, the mice were kept warm and monitored during recovery from anesthesia.

### Tissue Collection and Lung Leukocyte Isolation

The procedures were performed as previously described with modifications (19). At the time of data collection, the mice were sacrificed and perfused with 5 ml sterile PBS. The lungs were removed, minced with scissors, and added to homogenization gentleMACS C tubes containing proprietary catalysts for mechanical and enzymatic digestion (Miltenyi Biotec, Auburn, CA, USA). This process was followed by lung tissue homogenization using the gentle MACS dissociator (Miltenyi Biotec) and incubation at 37°C for 30 min in 4 ml/mouse digestion buffer (RPMI 1640, 5% fetal bovine serum, penicillin, and streptomycin, Invitrogen, Grand Island, NY, USA), 1 mg/ml collagenase A (Roche Diagnostics, Indianapolis, IN, USA), and 30 g/ml DNase I (Sigma, St. Louis, MO, USA). Erythrocytes were removed using 1× RBC lysis buffer (eBioscience, San Diego, CA, USA). Homogenized tissue was then passed through a 70 μm cell strainer (BD Falcon, Bedford, MA, USA) and centrifuged at 300 × *g* for 10 min to pellet the cells. The filtrate was centrifuged for 25 min at 1,500 × *g* in the presence of 20% Percoll (Sigma, St. Louis, MO, USA) in complete RPMI 1640 medium (RPMI 1640, 5% FBS, penicillin, and streptomycin) with no brake to separate leukocytes from cell debris and epithelial cells. Leukocyte pellets were resuspended in 5 ml complete RPMI 1640 medium and counted in a hemocytometer using trypan blue staining to exclude dead cells.

### Tissue CFU Assay

For determining the fungal burden in the lungs, brains, and spleens, tissues were removed and homogenized in 1 ml sterile PBS. Ten-fold dilutions of the samples were plated in duplicate on Sabouraud dextrose agar plates. Colonies were counted after 48 h of growth at 30°C, and CFU were calculated on a per-gram basis.

### Cytokine Analysis

Mouse serum was obtained from blood samples collected by heart puncture before lung excision and centrifugation at 10,000 × *g* for 10 min. Homogenates of lungs were centrifuged, and supernatants were diluted for cytokine analysis. Leukocytes isolated from mice lung were plated at 10^7^ cells/ml, and supernatants were collected by centrifugation of the culture medium. Mouse TNFα, IFNγ, IL-1α, IL-1β, IL-4, IL-6, IL-12, IL-17A, and IL-33 ELISA kits were from eBioscience. CXCL1 was from Raybio (Norcross, GA, USA).

### Flow Cytometry Analysis of Leukocyte Populations

For the flow cytometry experiments, antibodies were purchased from eBioscience, BioLegend, or BD Biosciences, including anti-murine CD16/CD32; CD45 conjugated to PerCP-Cy5.5; CD3, CD193, CD80, IFNγ, and annexin V conjugated to FITC; CD4, Ly6G, and Siglec F conjugated to APC; CD8, CD11c, CD19, and CD80 conjugated to PE-Cy7; CD19, CD40, F4/80, and IL-4 conjugated to PE; CD11b, CD326, and MHC II conjugated to APC-Cy7; and CD19, CD80, and Ly6C conjugated to BV421.

Leukocytes were isolated from the lung and lymph nodes of mice. Cell surface immunofluorescence staining involved the addition of a fluorochrome-conjugated antibody mixture containing antibodies specific to various leukocyte subpopulations to the staining buffer. Cells were incubated on ice for 30 min in the dark and washed twice with PBS. For intracellular cytokine staining, cells were fixed in IC fixation buffer and stimulated with cell stimulation cocktail (plus protein transportation inhibitors) from eBioscience. Cells were resuspended in permeabilization buffer (eBioscience) and stained with intracellular antibody cocktail. After staining, cells were immediately analyzed by flow cytometry (FACSAria III, BD Biosciences). FlowJo (For Mac OS X, version X 10.0.7r2, Tree Star, San Carlos, CA, USA) was used for data analysis. Leukocyte populations were identified using the following markers as previously described (20, 21): neutrophils (CD45^+^ Ly6G^+^ CD11b^+^), dendritic cells (DCs, CD45^+^ CD11c^+^ MHC II high), resident macrophage (CD45^+^ CD11b^−^ Siglec F^+^), eosinophils (CD45^+^ CD11b^+^ Siglec F^+^), monocyte-derived DCs or macrophages (CD45^+^ Ly6C^+^ CD11c^−^), CD4 T cells (CD45^+^ CD3^+^ CD4^+^), CD8 T cells (CD45^+^ CD3^+^ CD8^+^), and B cells (CD45^+^ CD19^+^). DCs in lymph nodes were stained with extracellular CD45, CD11c, and CD80, and intracellular TNFα, IFNγ, and IL-4. Total numbers of each cell population were calculated by multiplying the frequency of the population by the total number of leukocytes (the percentage of CD45^+^ cells multiplied by the original hemocytometer counts for total cells).

### Lung-Associated Lymph Node (LALN) Leukocyte Isolation

Lung-associated lymph node leukocytes were collected as previously described with modifications (22). Lymph nodes were removed from the mediastinum and then mechanically dispersed using an 1-ml sterile syringe plunger to press them through a 70 μm cell strainer (BD Falcon, Bedford, MA, USA) in complete medium. After centrifugation at 2,500 × *g* for 5 min, the supernatant was removed and the cell pellets saved for further use.

### Immunoblot Analysis

Lungs were ground up in liquid nitrogen and suspended in lysis buffer containing Tris–HCl (50 mM; pH 8.0), NaCl (150 mM), EDTA (1 mM), NP-40 (1%), PMSF (1 mM; Sigma), phosphatase inhibitor (Sigma), and a protease inhibitor cocktail (Roche Biochemical Laboratories). After incubation on ice for 30 min, the cell lysates were collected after centrifugation (14,000 × *g* for 10 min) at 4°C, and protein concentrations were determined using the Pierce BCA Protein Assay Kit (Thermo Scientific). A total of 30 μg protein was loaded for western blot analysis using the following antibodies: RIPK3 (Prosci) and caspase-3 (Cell Signaling Technology).

### Real-time PCR Analysis

Total RNA (10–100 ng, depending on the abundance of the target gene) was purified using TRIzol reagent (Ambion by Life Technologies) for RT-qPCR in a one-step reaction with Reverse Transcriptase (Takara) and SYBR green master mix (Takara) using a 7900 Real-Time PCR system (Applied Biosystems). All primers used for RT-qPCR are listed in Table S1 in Supplementary Material. The qPCR analysis was performed using the 2^−ΔCt^ method, and target genes were normalized to the housekeeping genes in each strain.

### Histology, Immunohistochemistry, and Immunofluorescence

Lungs were instilled with 1 ml 10% neutral-buffered formalin, excised, immersed in 10% neutral-buffered formalin, and embedded in paraffin as described previously (19). Five-micrometer sections were cut and stained with hematoxylin & eosin. Immunohistochemical and immunofluorescent staining was performed using formalin-fixed, paraffin-embedded tissue sections with rabbit anti-RIPK3 (Prosci) antibodies. Sections were photographed by Zeiss light microscopy (ZEISS, AXIO) and Olympus confocal microscopy (FV1000). Lung tissue inflammation and injury score were performed by three different pathologists in a blinded fashion. The quantify criteria is referenced to previous studies with modification (23).

### Fungal Killing and Cell Viability Assays

Bone marrow-derived macrophages (BMDMs) were generated as previously described (24, 25). Briefly, marrow was flushed from the C57Bl/6 mouse femurs and tibias and dispersed into a single-cell suspension. The cells were cultured for 7 days in RPMI medium supplemented with 10% FBS and 50 ng/ml M-CSF. The cultures were additionally nourished with M-CSF-containing medium on the third day of culture. All *in vitro* experiments were performed in RPMI 1640 containing 10% FCS and 5 ng/ml M-CSF.

For the fungal killing assay, freshly isolated BMDMs were diluted to 10^6^ cells/ml and plated on a 96-well cell culture plate. *C. neoformans* were washed twice with PBS, resuspended in RPMI medium, and adjusted to 10^5^ cells/ml. The yeast cells were further opsonized with anti-GXM antibody for 1 h at 37°C, followed by the addition of 100 μl opsonized *C. neoformans* to each well of the BMDM culture plate and incubation at 37°C with 5% CO_2_ for 24 h. BMDM cells were lysed in sterile water for 20 min, mixed with the supernatant, and then diluted and plated on Sabouraud agar plates. CFU were counted after 2 days at 30°C.

### Statistical Analysis

All data are expressed as means ± SEMs. The data obtained for the animal survival assays were plotted as Kaplan–Meier survival curves and analyzed with the log-rank test using GraphPad Prism version 6.00 for Windows (GraphPad Software, San Diego, CA, USA). The remaining statistical analyses were conducted with the ANOVA, Student’s *t*-test, and Kruskal–Wallis test as well as Dunn’s test for non-parametric measures. The results were considered statistically significant when the *P* value was less than 0.05.

## Results

### RIPK3 and FADD Critically Contribute to Host Defenses against *C. neoformans* Infection

**Figure 1:**
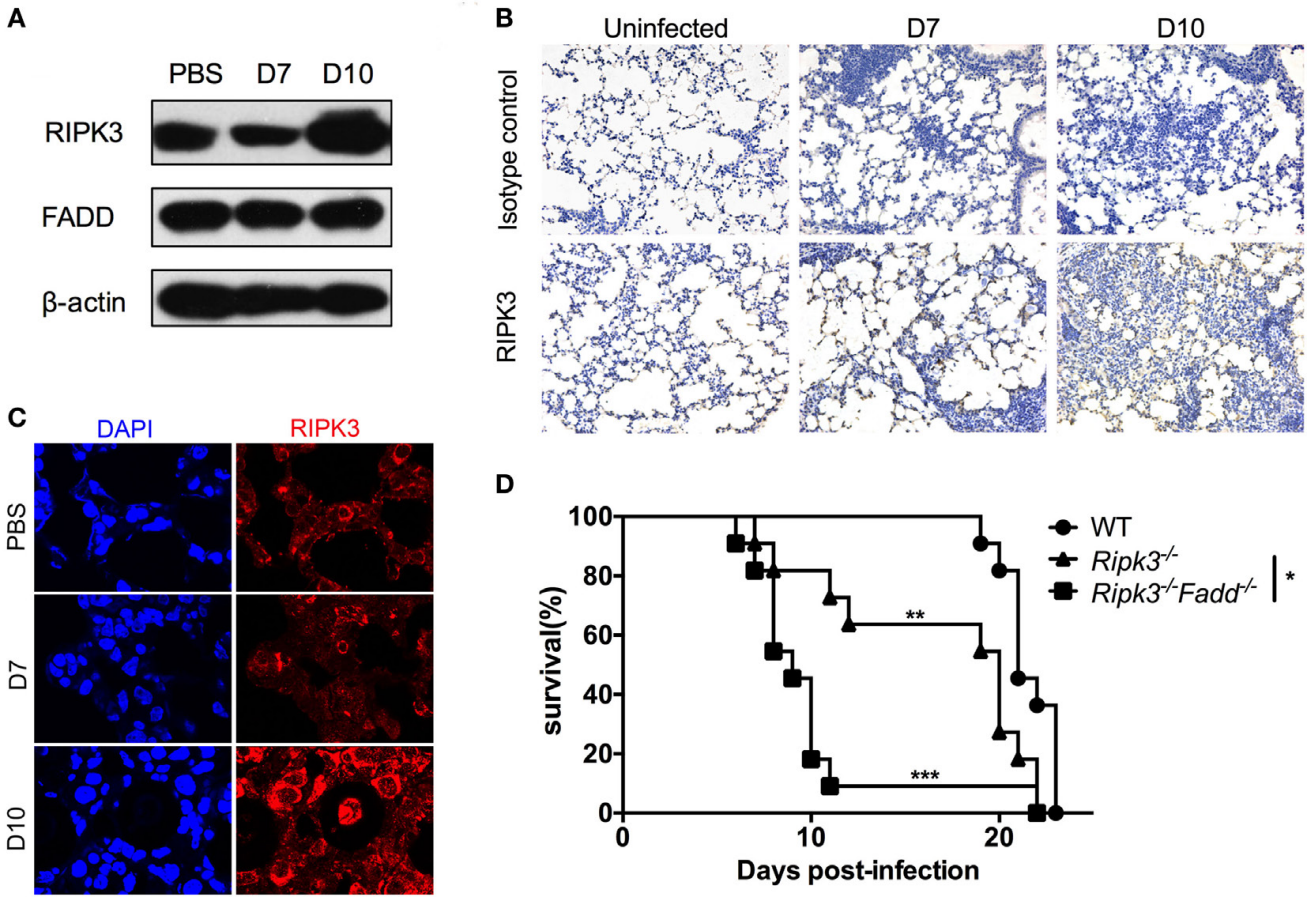
RIPK3 and Fas-associated death domain (FADD) critically contribute to host defenses against *Cryptococcus neoformans* infection. C57BL/6 mice were inoculated intranasally with 10^5^ *C. neoformans* strain H99 and sacrificed at 7 or 10 days postinfection. PBS-treated mice were utilized as control. Pulmonary expression of RIPK3 and FADD were examined by western blot analysis in total protein form lung homogenates **(A)**. RIPK3 displayed enhanced expression, while FADD remains unchanged in the lung of infected mice. Fungal infection significantly enhanced the local recruitment of RIPK3 in the lung as shown by immunohistochemistry **(B)** and immunofluorescence **(C)** assays. Wild-type (WT), *Ripk3*^−/−^, *Ripk3*^−/−^*Fadd*^−/−^ mice (*n* = 11 for each group) were infected *via* inhalation to determine effects of RIPK3 and FADD in host defenses against *C. neoformans*. Survival studies showed that *Ripk3*^−/−^ and *Ripk3*^−/−^*Fadd*^−/−^ mice were more susceptible to fungal infection **(D)**. *P < 0.05, **P < 0.01, ***P < 0.001. The survival study was repeated three times independently.

To gain insight into the roles of RIPK3 and FADD during host responses to *C. neoformans* infection, we first evaluated kinetics of RIPK3 and FADD protein expression in the lungs of the infected C57BL6 mice. Immunoblot study results showed that RIPK3 displayed extensive upregulation in the lung tissue at 10 days postinfection (dpi) (Figure 1A), further verified by immunochemistry and immunofluorescence assays (Figures 1B,C). In contrast, FADD, albeit abundant, appeared to be expressed constitutively throughout the studied time points of infection. Next, to elucidate the relative importance of FADD and RIPK3-related signaling in anti-cryptococcal defense, we compared the survival of infected *Ripk3*^−/−^, *Ripk3*^−/−^*Fadd*^−/−^, and WT mice. As shown in Figure 1D, compared with WT mice, infected *Ripk3*^−/−^ mice showed accelerated onset of mortality (8 vs 20 dpi) and reduced median survival (median: 16.5 ± 5.8 vs 21.5 ± 1.4 days). *Ripk3*^−/−^*Fadd*^−/−^ mice showed even greater susceptibility to *C. neoformans* infection compared with either *Ripk3*^−/−^ (*P* < 0.05) or WT mice (*P* < 0.001), exhibiting a drastically shortened median survival (9.9 ± 4.3 days). These results demonstrate that FADD and RIPK3 signaling critically contribute to host defense against cryptococcal infection, most likely in a synergistic fashion.

### RIPK3 or RIPK3/FADD Deletion Reduced Host Ability to Control Fungal Growth and Dissemination

**Figure 2:**
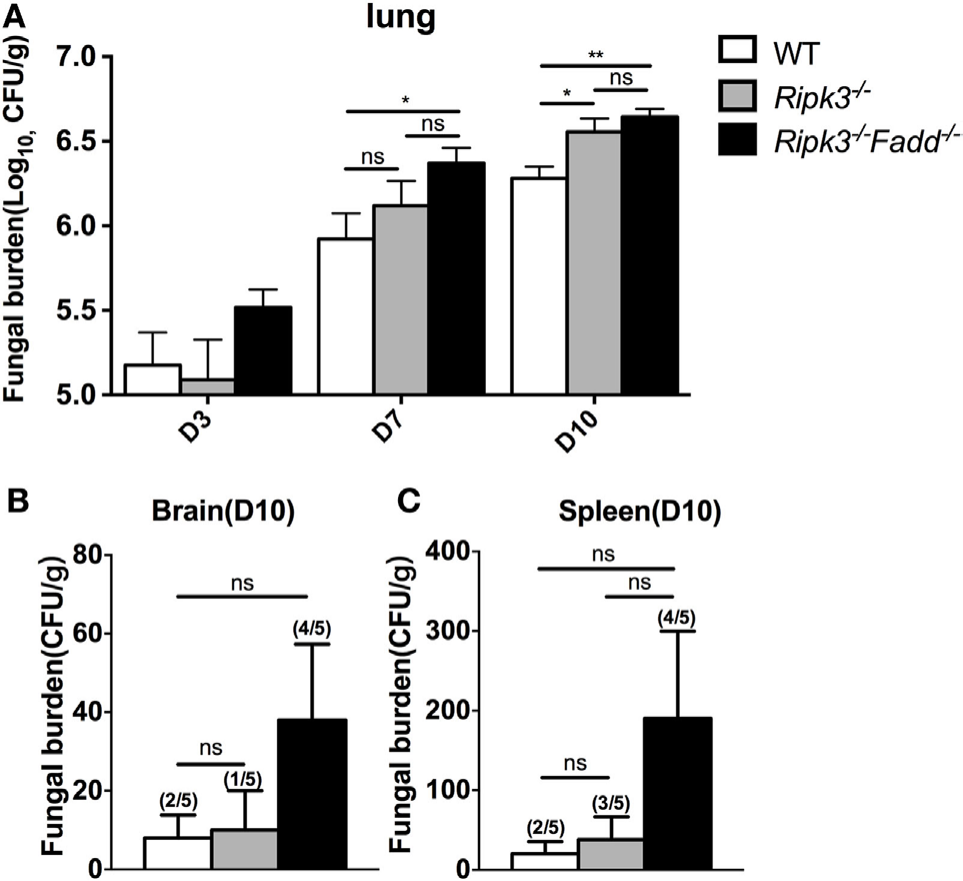
*Ripk3*^−/−^ and *Ripk3*^−/−^*Fadd*^−/−^ mice showed diminished fungal clearance. Pulmonary fungal burden assay revealed diminished fungal clearance of *Ripk3*^−/−^ and *Ripk3*^−/−^*Fadd*^−/−^ mice **(A)** and similar but not significant trends in extra-pulmonary dissemination **(B,C)**. Values represent means and SEMs (*n* = 5 mice for each group); ns, no significant difference, *P < 0.05, **P < 0.01, between compared groups. The colony-forming units (CFU) assay was repeated three times independently.

Next, we examined fungal burden in the lung, brain, and spleen in infected mice to assess the effects of RIPK3 and FADD in the control of fungal growth and systemic dissemination. Compared with WT, *Ripk3*^−/−^ mice had a significantly higher pulmonary fungal load at 10 dpi (*P* < 0.05), and RIPK3/FADD double deletions further enhanced the fungal burden in the lungs at both 7 dpi (*P* < 0.05) and 10 dpi (Figure 2A, *P* < 0.01). Similar trends in fungal burden were noted in the spleens and brains of *Ripk3*^−/−^ and *Ripk3*^−/−^*Fadd*^−/−^ mice at 10 dpi (Figures 2B,C), with frequencies of positive spleen and brain cultures in *Ripk3*^−/−^*Fadd*^−/−^ mice doubling those in the WT mice. Thus, both RIPK3 and FADD significantly contributed fungal containment during pulmonary cryptococcal infection.

### RIPK3/FADD Deletions Lead to Severe Lung Pathologies in *C. neoformans*-Infected Mice

**Figure 3:**
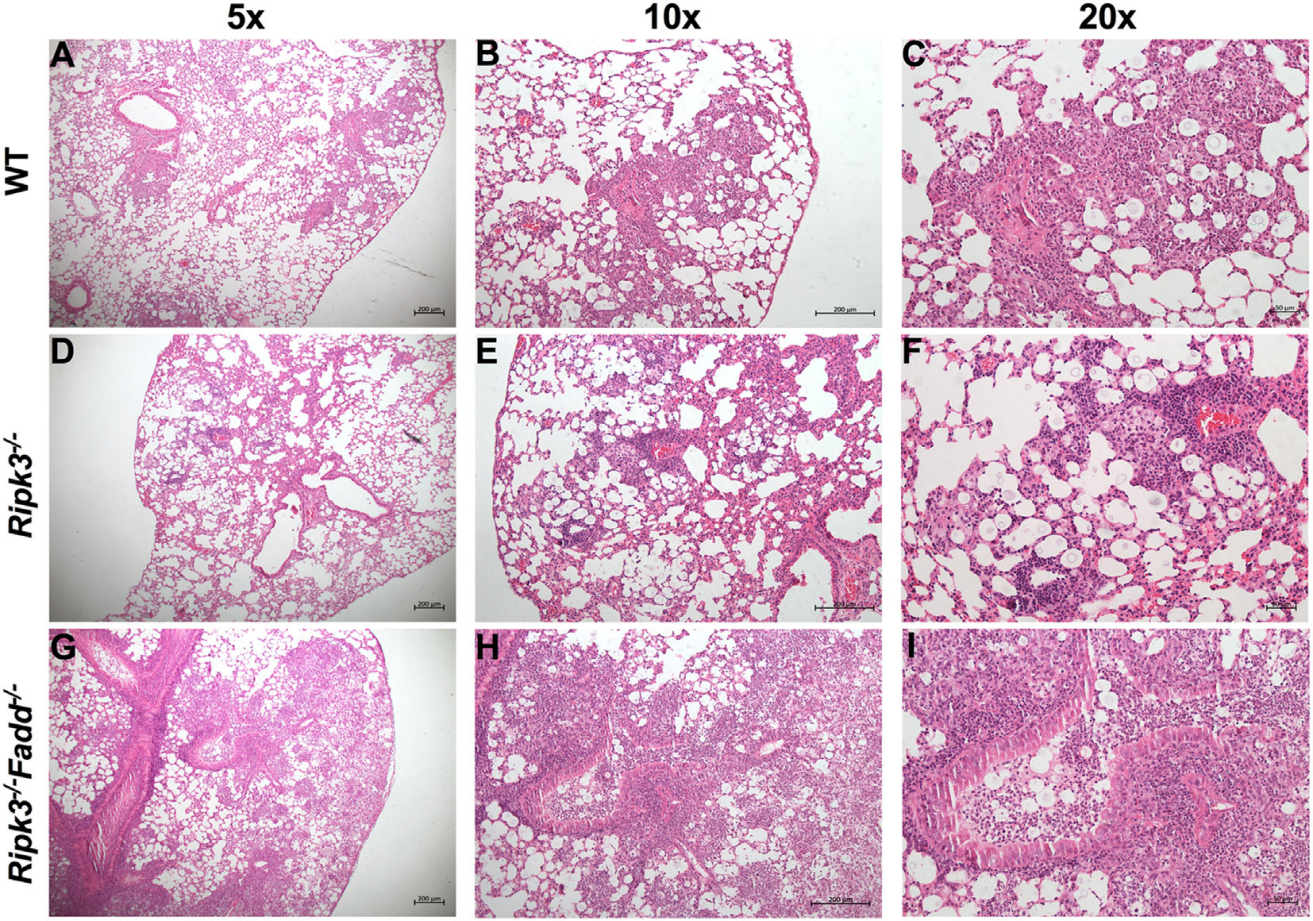
Deletion of RIPK3 and Fas-associated death domain (FADD) resulted in enhanced inflammatory infiltration and tissue injury following pulmonary infection with *Cryptococcus neoformans*. Wild-type (WT) **(A–C)**, *Ripk3*^−/−^ **(D–F)**, or *Ripk3*^−/−^*Fadd*^−/−^ **(G–I)** mice were infected with 10^5^ *C. neoformans* strain H99. At 10 days postinfection, lungs were harvested, and the morphological pattern of inflammation was evaluated by light microscopy. Pulmonary representative photomicrographs (hematoxylin and eosin stained) taken from each group were shown [**(A,D,G)** magnification of ×50; **(B,E,H)** magnification of ×100; **(C,F,I)** magnification of ×200]. Compared with WT group, *Ripk3*^−/−^ mice displayed similar pulmonary histological pattern but a slight enhancement in inflammatory infiltration. However, extensive pulmonary inflammation and severe tissue damage were observed in *Ripk3*^−/−^*Fadd*^−/−^ mice, indicating distinct patterns of lung pathology.

Rapid clinical deterioration and the accelerated mortality, especially in the infected *Ripk3*^−/−^*Fadd*^−/−^ mice could not be solely explained by relatively modest increases in fungal burdens at 7 and 10 dpi. Thus, we examined the effects of RIPK3 or RIPK3/FADD on the development of lung inflammation and pathologies post-*C. neoformans* infection. We examined lung sections obtained from sham-infected (PBS) and *C. neoformans*-infected WT and *Ripk3*^−/−^ or *Ripk3*^−/−^*Fadd*^−/−^ mice. Deletion of RIPK3 or RIPK3/FADD induced no visible alterations in uninfected lungs (data not shown), demonstrating that genetic defects in RIPK3 or RIPK3/FADD did not affect baseline pulmonary morphology. Comparative histopathological assessments of lung sections from each group at 10 dpi (Figure 3) demonstrated only subtle enhancement of pulmonary leukocyte infiltration in *Ripk3*^−/−^ mice relative to the control mice with largely similar pattern of inflammatory lesions (Figures 3A–C vs Figures 3D–F). The borders between inflamed regions and normal alveoli remained distinct at 10 dpi in WT and *Ripk3*^−/−^ mice (Figures 3A–F). Consistently, blinded pathology score, albeit showing an increasing trend, has not increased significantly (Figure S2 in Supplementary Material). In contrast, *Ripk3*^−/−^*Fadd*^−/−^ mice exhibited progressive pulmonary inflammation with severe tissue damage (Figures 3G–I). Less organized inflammatory infiltrates (predominantly neutrophils and lymphocytes) were spread diffusely through bilateral lung fields at 10 dpi. The margins of inflamed regions from uninvolved alveoli were less distinct. Features of suppurative bronchopneumonia, such as airway plugins with polymorphic neutrophil and dense cellular exudate, were observed throughout the lung (Figures 3H,I). The blinded pathology score showed that inflammation/pathology score was significantly greater in lungs of *Ripk3*^−/−^*Fadd*^−/−^ mice compared to both WT and *Ripk3*^−/−^ mice (Figure S2 in Supplementary Material), which corroborated well with survival data on day 10 (80% mortality in *Ripk3*^−/−^*Fadd*^−/−^ group, only 20% mortality in *Ripk3*^−/−^ group, and all mice surviving in the WT group at 10 dpi). Collectively, these findings demonstrated that concurrent RIPK3/FADD deletion during *C. neoformans* infection induced severe pulmonary inflammation and tissue damage, potentially explaining the highly accelerated mortality in *Ripk3*^−/−^*Fadd*^−/−^ mice.

### RIPK3 and FADD Differentially Modulated Pulmonary Leukocyte Accumulation during *C. neoformans* Infection

**Figure 4:**
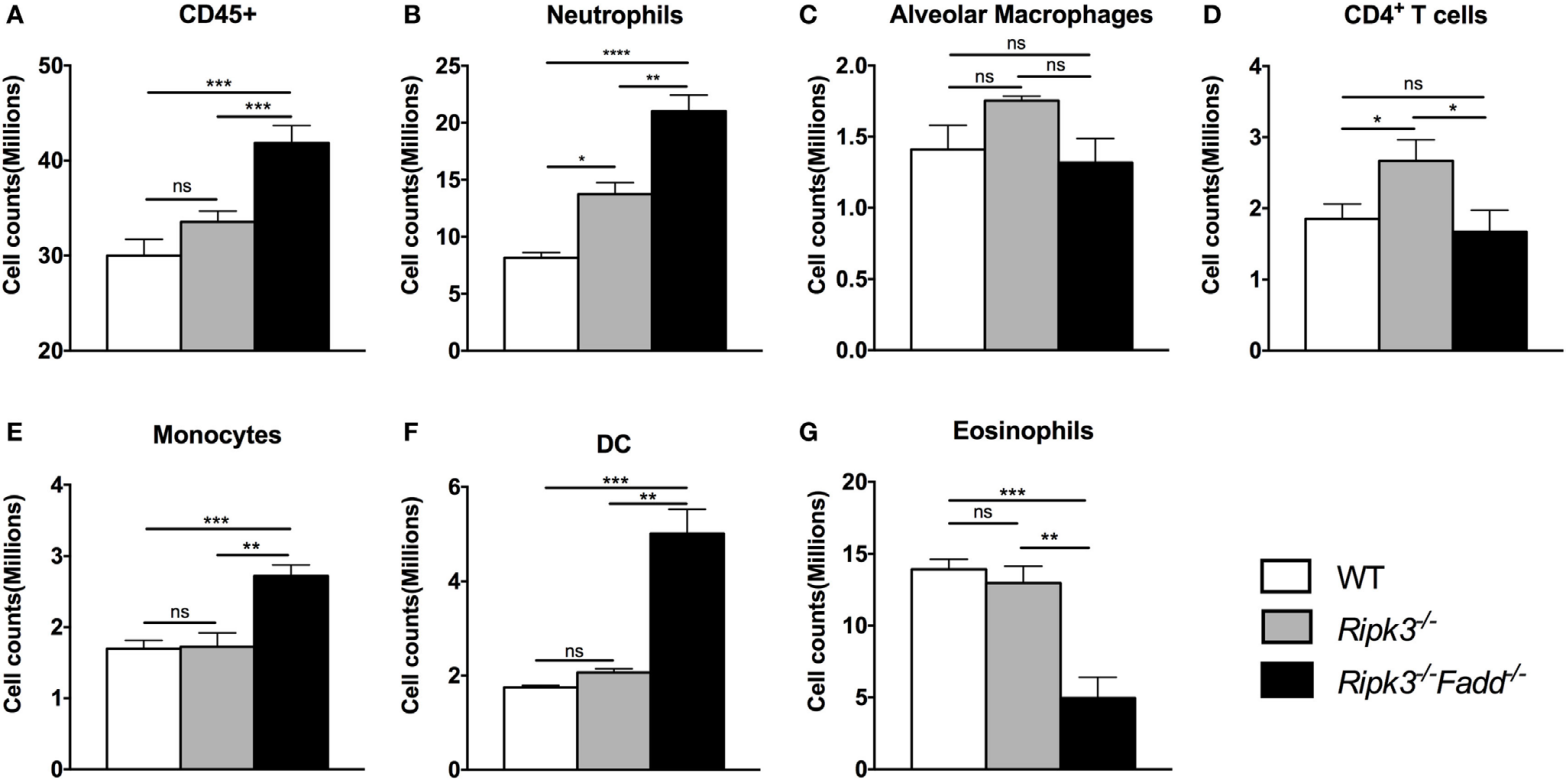
RIPK3 or RIPK3/Fas-associated death domain (FADD) deletions resulted in alteration in pulmonary leukocyte recruitments. Mice were inhalationally infected with 10^5^ *Cryptococcus neoformans* strain H99. Lungs were harvested at 10 days postinfection, and total pulmonary leukocytes were isolated from infected lungs after enzymatic digestion. Magnitude and/or leukocyte composition were then analyzed by flow cytometry. Results are illustrated as absolute numbers of each lymphocyte subset (**A**–**G**) in the total lung sample. Values represent means and SEMs (*n* = 6 mice per group). ns, no significant difference, *P < 0.05, **P < 0.01, ***P < 0.001, ****P < 0.0001, between compared groups. This experiment was repeated twice.

To quantify the effects of RIPK3 and FADD deletions on cellular components of the inflammatory response to *C. neoformans*, we compared leukocyte populations isolated from uninfected or infected lungs of WT, *Ripk3*^−/−^, or *Ripk3*^−/−^*Fadd*^−/−^ mice at 10 dpi. Consistent with the histopathological findings, flow cytometric analysis revealed no significant effect of *Ripk3*^−/−^ or *Ripk3*^−/−^*Fadd*^−/−^ mutations in uninfected mice for any of the leukocyte subsets (data not shown). While only a borderline increase in total lung leukocytes counts (CD45^+^ cells) was observed in *Ripk3*^−/−^ mice compared to WT mice at 10 dpi (Figure 4A, *P* < 0.06), significant increases in two pulmonary leukocyte subsets were observed. *Ripk3*^−/−^ mice showed increased numbers of neutrophils (Figure 4B), increasing trend in macrophages (Figure 4C), and elevated CD4^+^ T cells (Figure 4D) compared to the infected WT mice.

*Ripk3*^−/−^*Fadd*^−/−^ mice infected by *C. neoformans* showed more profound alterations in both the magnitude of inflammation and leukocyte composition. There was a significant increase in total leukocyte counts in *Ripk3*^−/−^*Fadd*^−/−^ mice compared with WT group at 10 dpi (Figure 4A), mostly driven by a remarkable increase in neutrophil numbers (more than threefold) relative to infected WT (Figure 4B) and significantly greater than in the *Ripk3*^−/−^ mice. Significant increases in other myeloid cell subsets (monocytes, Figure 4E; DCs, Figure 4F) were also detected relative to both WT and *Ripk3*^−/−^ mice. However, the numbers of pulmonary eosinophils observed in abundance in the WT and *Ripk3*^−/−^ mice at 10 dpi were suppressed in *Ripk3*^−/−^*Fadd*^−/−^ mice (Figure 4G), suggesting a shift away from a Th2 response in the WT following RIPK3/FADD double deletion. Collectively, these data further support that RIPK3 and more so the RIPK3/FADD double deletion results in an exuberant accumulation of inflammatory cells and immunomodulation that alters the course of inflammatory response implicated in lung injury we observe during *C. neoformans* infection in the absence of these factors.

### RIPK3/FADD Deletion Reshaped the Cytokine Responses during Pulmonary *C. neoformans* Infection

**Figure 5:**
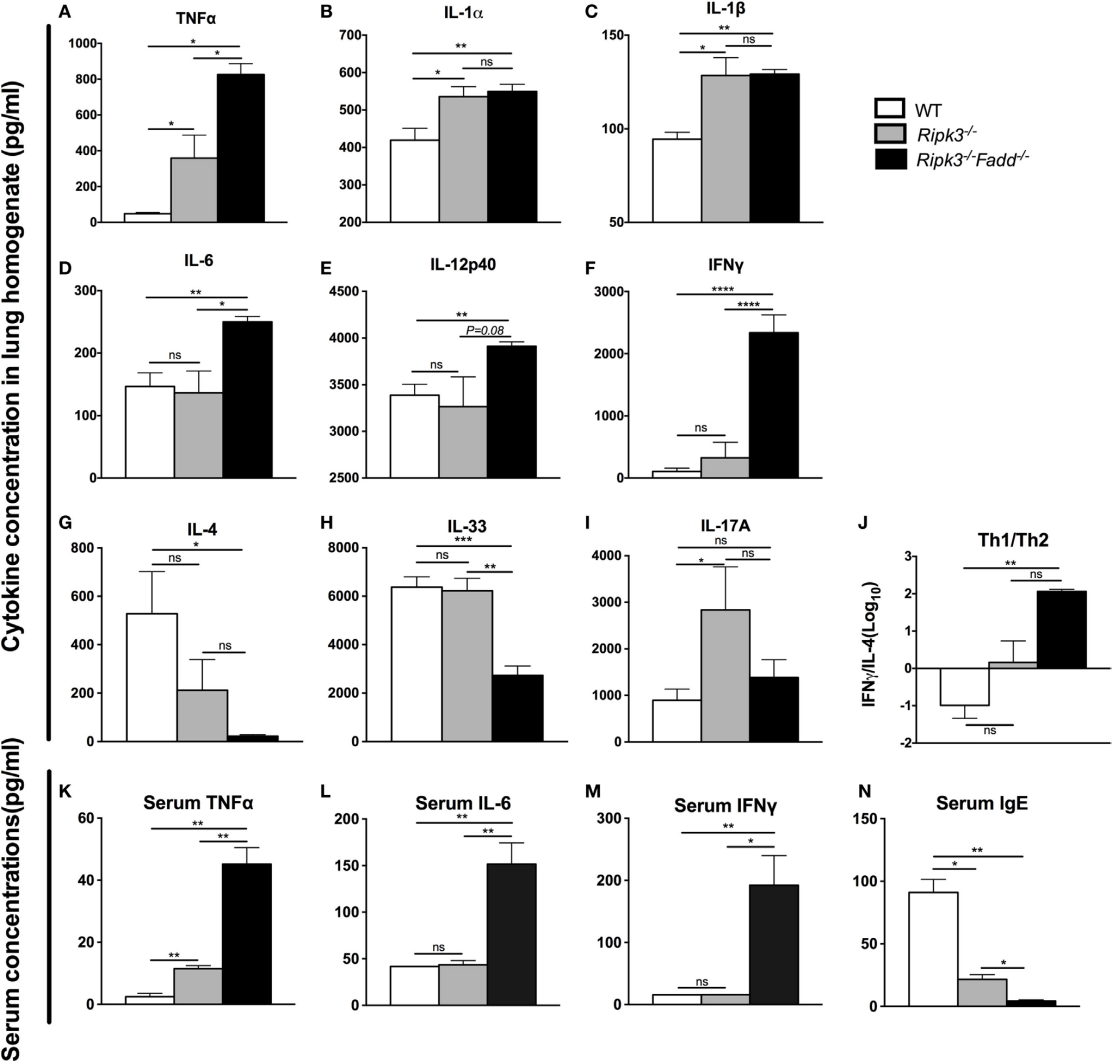
RIPK3 or RIPK3/Fas-associated death domain (FADD) deletions altered Th-polarizing and pro-inflammatory cytokine profiles in local (pulmonary) and systemic response to cryptococcal infection. Total lung tissue **(A–I)** and serum **(K–N)** were harvested from wild-type (WT), *Ripk3*^−/−^, or *Ripk3*^−/−^*Fadd*^−/−^ mice at 10 days postinfection with *Cryptococcus neoformans*. Supernatants of lung tissue homogenates were collected by centrifuging. Cytokine levels were detected in the supernatants and serum by ELISA. Total serum IgE concentrations were also measured by ELISA. Values represent mean cytokine or IgE concentrations [**(A–I)**, **(K–N)**, pg/ml] or **(J)**, the IFNγ/IL-33 ratio (*n* = 5 mice per group). ns, no statistical significance. *P < 0.05, **P < 0.01, ***P < 0.001. This experiment was repeated twice.

Having demonstrated that RIPK3 and RIPK3/FADD deletions altered inflammatory infiltrate compositions, we further examined the roles of RIPK3 and FADD in pulmonary and systemic cytokine levels during cryptococcal infection. Lung homogenates and serum isolated at 10 dpi from *Ripk3*^−/−^, *Ripk3*^−/−^*Fadd*^−/−^, and WT mice were analyzed by ELISA (Figures 5A–N). We found that depletion of RIPK3 alone significantly increased pro-inflammatory cytokine production: TNF-α, IL-1α, and IL-1β (Figures 5A–C). Non-significant increasing trend in IFN-γ level and decreasing trend in IL-4 production suggested cytokine profile drifting away from Th2 to Th1 pattern (Figure 5J), which corresponded to diminished serum IgE accumulation (Figure 5N). However, no difference in IL-12p40 or IL-33 have been observed (Figures 5E,H), suggesting that the full switch from Th2 to Th1 has not occurred as a result of RIPK3 deletion. Finally, while we observe significant increase in IL-17A, which might have suggested shift toward Th17 response as a result of RIPK3 deletion, there was no concurrent increase in IL-6 or IL-12p40, which would be associated with Th17 response. Most of these trends were reproduced in pulmonary leukocyte cell culture supernatants (Figure S4 in Supplementary Material). Consistently, with lung cytokines, serum cytokine analysis showed somewhat elevated TNFα (Figure 5K), suggesting more pronounced inflammatory response levels in infected *Ripk3*^−/−^ mice, but no increase in serum IL-6 or IFNγ (Figures 5L,M).

RIPK3/FADD double deletion further enhanced pro-inflammatory TNFα and IL-6 (Figures 5A,D) and showed sustained elevation of IL-1α, IL-1β compared to the WT mice. Furthermore, Th1 cytokines IL-12p40 and IFNγ were significantly elevated relative to WT and RIPK3 mice levels of these cytokines (Figures 5E,F), suggesting not only strongly intensified inflammatory response but also shift to a Th1. Consistently, Th2 cytokines (IL-4 and IL-33) were profoundly suppressed (Figures 5G,H), and the Th1/Th2 ratio increased more than 300-fold in RIPK3/FADD-depleted relative to WT mice (Figure 5J), with corresponding absence of serum IgE accumulation (Figure 5N) strongly suggesting the development of a robust Th1 bias in these mice. Interestingly, IL-17 was not elevated as in the *Ripk3*^−/−^ mice but showed level similar to that in the infected WT mice (Figure 5I).

**Figure 6:**
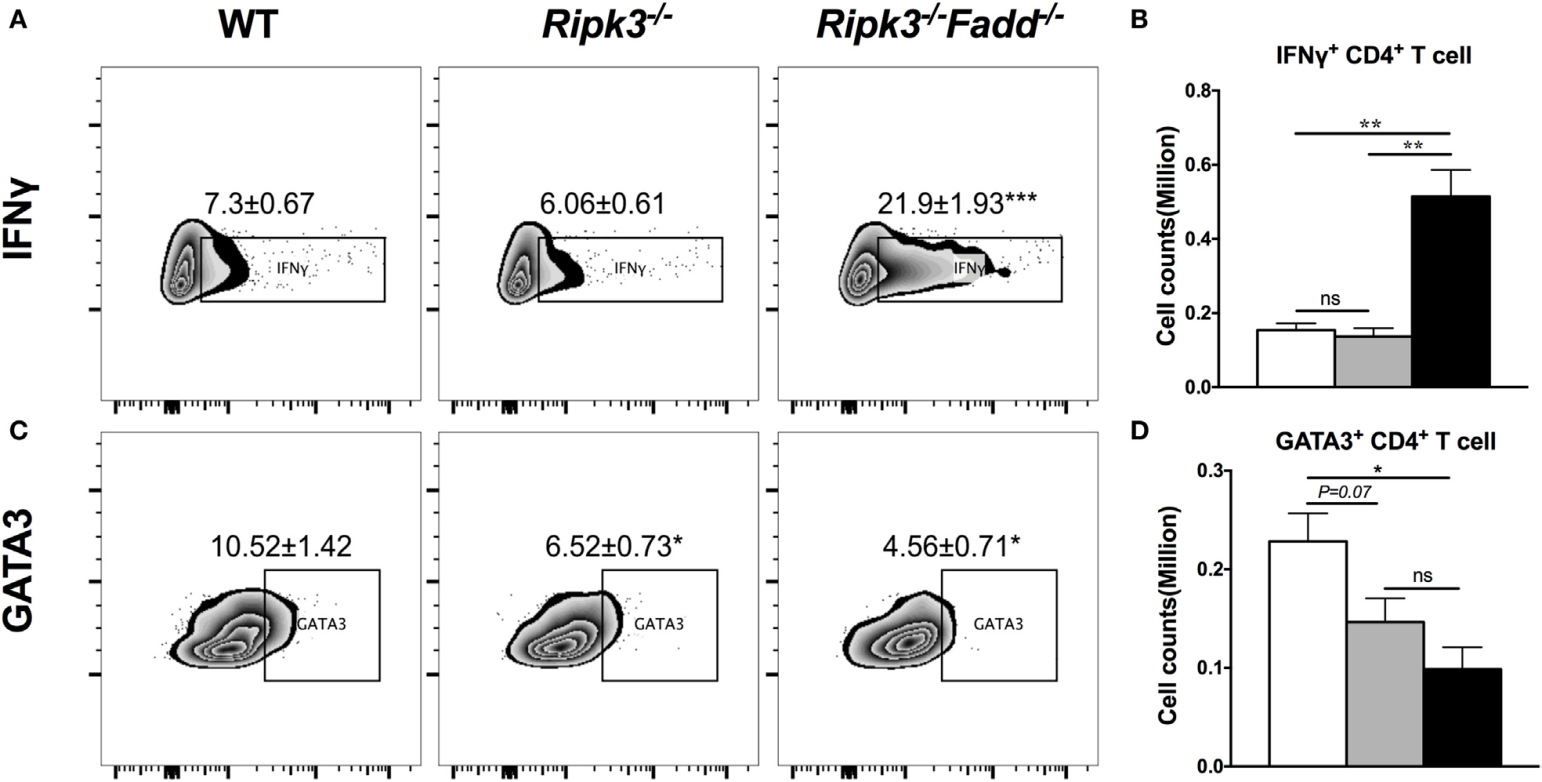
RIPK3 or RIPK3/Fas-associated death domain (FADD) deletions affects Th1/Th2 polarization of CD4 T cells in the lungs of *Cryptococcus neoformans*-infected mice. Leukocytes were isolated from the lungs of infected mice on 9 days postinfection. Intracellular expression of IFNγ, GATA3 in CD4 T cell population was analyzed by flow cytometry. **(A,C)** show the percentage of IFNγ or GATA positive populations in CD4^+^ cells. **(B,D)** show the cell counts of IFNγ or GATA positive CD4 T cells. Note that there was a significant increase of Th1 markers in the *Ripk3*^−/−^*Fadd*^−/−^ mice compared to the control mice **(A,B)**. Results represent means and SEMs (*n* = 4 mice for each time point). *P < 0.05, **P < 0.01.

To further investigate effects of RIPK3/FADD on T cell polarization, we performed intracellular flow analysis on pulmonary CD4^+^ T cells from infected *Ripk3*^−/−^ *and Ripk3*^−/−^*Fadd*^−/−^ mice at 10 dpi. Consistent with the cytokine data, percentage of IFNγ^+^ CD4^+^ T cells was not different in *Ripk3*^−/−^ mice compared to the WT, but increased in *Ripk3*^−/−^*Fadd*^−/−^ mice compared to WT and *Ripk3*^−/−^ mice (Figures 6A,B). Moreover, we found there was a borderline reduction in the frequencies of GATA3 positive CD4^+^ T cells in *Ripk3*^−/−^ (*P* = 0.07) mice and significant decrease in frequencies of GATA3^+^ CD4^+^ T cells in *Ripk3*^−/−^*Fadd*^−/−^ mice relative to WT mice at 10 dpi (Figures 6C,D). Interestingly, no increase in IL-17A or RorγT^+^ CD4 or CD8 T-cells was observed in either *Ripk3*^−/−^ or *Ripk3*^−/−^*Fadd*^−/−^ mice compared to the WT mice (data not shown). Collectively, analysis of cytokine responses and T-cell polarization profile showed that RIPK3 single deletion led to enhanced pro-inflammatory responses, while that additional deletion of FADD further potentiated these effects, leading to a very strong Th1 bias systemic and “cytokine storm” at 10 dpi with *C. neoformans*.

### RIPK3 and FADD Deletions Potentiated Classical Activation of Macrophages and Their Fungicidal Responses *In Vitro*

**Figure 7:**
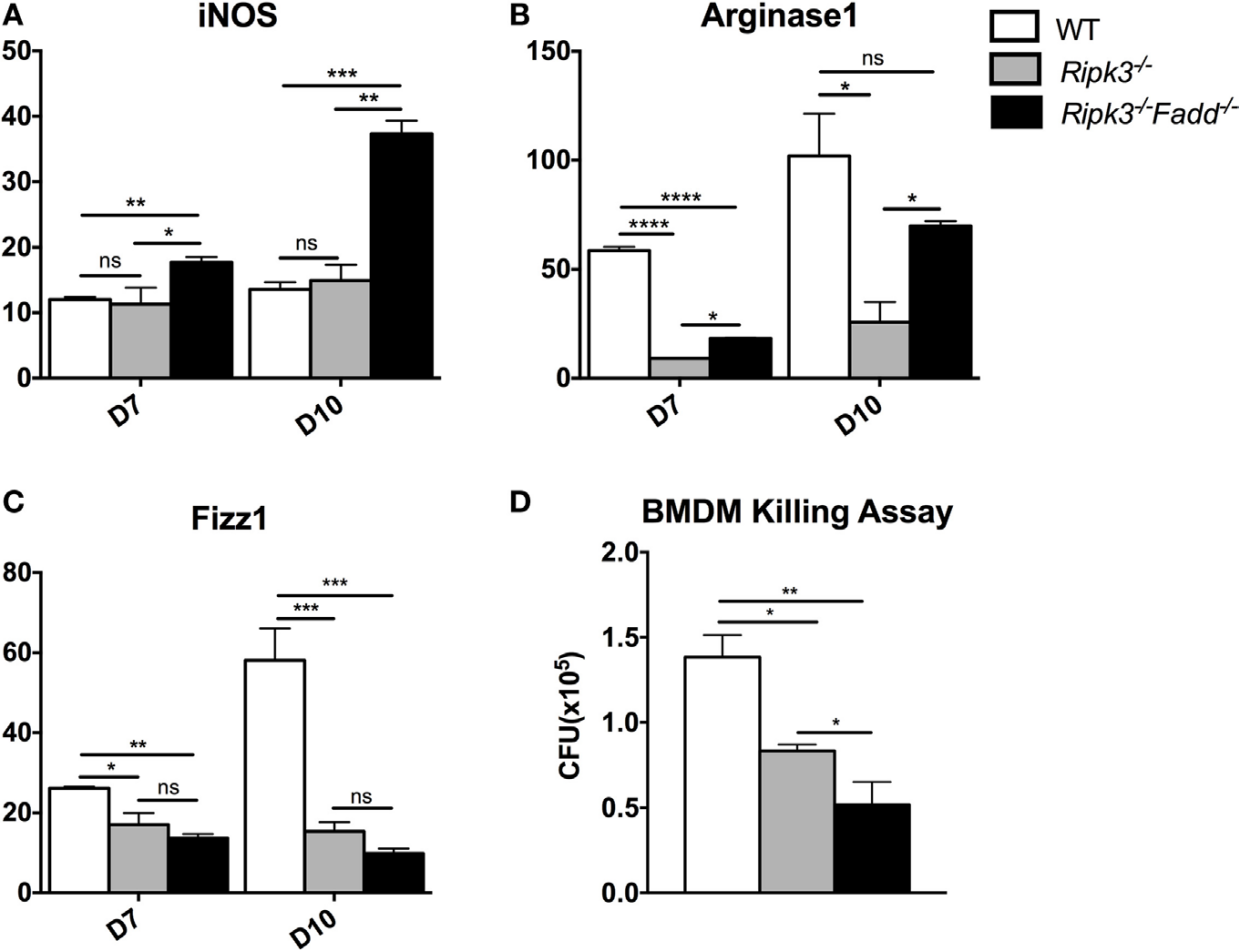
*Ripk3*^−/−^ and *Ripk3*^−/−^*Fadd*^−/−^ mice displayed progressively classical activation and increased fungicidal ability of macrophages. mRNA was isolated from pulmonary macrophages of WT, *Ripk3*^−/−^, or *Ripk3*^−/−^*Fadd*^−/−^ mice at 10 days postinfection. Real-time PCR was performed to determine the expression levels of iNOS [nitric oxide synthase, **(A)**], Arg1 [arginase 1, **(B)**], and Fizz1 **(C)**. Bone marrow-derived macrophages (BMDMs) isolated from uninfected mice were utilized to detect intracellular fungicidal ability **(D)**. ns, no significant difference, *P < 0.05, **P < 0.01, ***P < 0.001, ****P < 0.0001, between compared groups. These experiments were repeated three times.

Macrophages are distal effector cells that execute anti-*C. neoformans*-based cytokine responses in infected organs (26, 27). Having determined that RIPK3 increased inflammatory cytokines, including pro-M1 cytokine TNFα and RIPK3/FADD additionally promoted Th1 responses, we assessed the M1/M2 polarization patterns of macrophages in the infected lungs and the fungicidal ability of BMDMs isolated from uninfected mice from each strain. The expression of M1- and M2-associated genes was evaluated by real-time PCR (Figures 7A–C). Consistent with absence of major increase in IFNγ production, *Ripk3*^−/−^ mice macrophages did not show significant upregulation of the M1 activation marker iNOS (Figure 7A). However, in concert with less pronounced Th2 and more pro-inflammatory environment in the lungs, we observed diminished upregulation of M2 markers arginase Arg1 and Fizz1 (Figures 7B,C), suggesting that macrophages in the infected lung of *Ripk3*^−/−^ mice were less M2 biased. Consistent with the strong Th1-type polarization, expression of the iNOS was significantly upregulated in *Ripk3*^−/−^*Fadd*^−/−^ mice (Figure 7A), while Fizz1 expression was diminished (Figure 7C) compared to both WT and *Ripk3*^−/−^ mice. Compared with WT mice, arginase1 expression in *Ripk3*^−/−^ *Fadd*^−/−^ mice also showed downward effects (Figures 7B,C). To determine whether the fungicidal potential (typically linked to M1/M2 activation) was affected in *Ripk3*^−/−^ and *Ripk3*^−/−^*Fadd*^−/−^ mice, we evaluated the fungicidal effect of the BMDMs after co-incubation with 10^6^ CFU of *C. neoformans* (Figure 7D). Significantly reduced cryptococcal survival was observed in *Ripk3*^−/−^ and, to an even greater extent, in *Ripk3*^−/−^ *Fadd*^−/−^ macrophages, further demonstrating that, at the cellular level, RIPK3 and FADD deletions promoted rather than suppressed fungicidal M1 macrophage polarization. Thus, the effects of RIPK3 and FADD deletion on M1/M2 gene expression were consistent with the cytokine profiles in the *C. neoformans*-infected lungs.

### RIPK3 and FADD Deletions Promoted DC Differentiation and Activation in LALNs after Cryptococcal Infection

**Figure 8:**
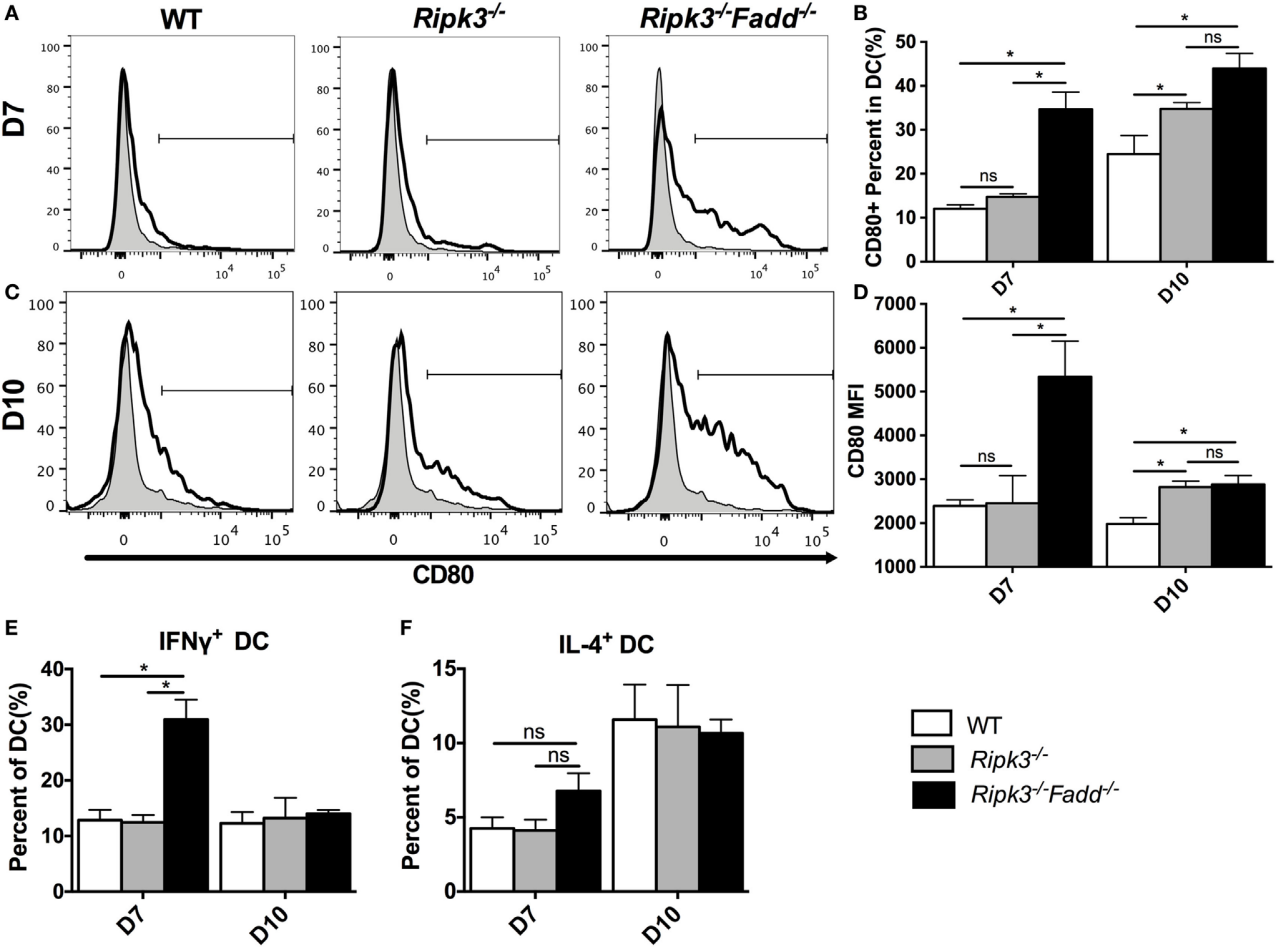
RIPK3 and Fas-associated death domain (FADD) deletion promoted dendritic cells differentiation and activation in lung-associated lymph nodes (LALNs) after cryptococcal infection. LALNs were harvested from wild-type (WT), *Ripk3*^−/−^, and *Ripk3*^−/−^*Fadd*^−/−^ mice at 7 and 10 days postinfection. Dendritic cells (DCs) were stained with CD80 **(A–D)**, IFNγ **(E)**, and IL-4 **(F)** antibodies and then analyzed by flow cytometry. Data are expressed as means and SEMs (*n* = 3 or more mice per group). ns, no significant difference, *P < 0.05, between compared groups. This experiment was repeated twice.

Having determined that joint RIPK3/FADD deletion promoted a strong shift toward the Th1/M1 response in contrast with single RIPK3 deletion, we analyzed the phenotype of DCs, the central regulators of Th-immune polarization. Flow cytometric analysis of LALN DC was performed to examine the frequency and intensity of CD80, a major co-stimulatory molecule expressed during DC co-stimulatory maturation, and the DC1 phenotypic marker and predictor of Th1 response development (22, 28, 29). Consistent with strong Th1 bias, the frequency and intensity of CD80 expression was significantly enhanced in LALN DC of *Ripk3*^−/−^*Fadd*^−/−^ mice at both 7 and 10 dpi (Figures 8A–D) compared to those in the infected WT mice. *Ripk3*^−/−^ mice also did not show this effect on day 7 but displayed some increase of CD80 surface expression at 10 dpi (Figures 8C,D). Cytokine expression in LALN DCs was also analyzed by intracellular staining. Again, notable enhancement of IFNγ expression was observed in LALN DCs of *Ripk3*^−/−^*Fadd*^−/−^ mice at day 7 but without significant alteration in IL-4 production (Figures 8E,F). However, RIPK3 deletion had no effect on LALN DC cytokine expression. Taken together, these results suggested that RIPK3/FADD deletions synergistically promoted LALN DC maturation and DC1 activation during *C. neoformans* infection, which was consistent with strongly enhanced Th1 responses in these mice, while changes in *Ripk3*^−/−^ DC activation profile was subtle, consistent with subtle effects of *Ripk3*^−/−^ Th polarization profile during *C. neoformans* infection.

### RIPK3 and FADD Deletions Differentially Affected Apoptosis Rate in Leukocyte Subsets during *C. neoformans* Infection

To further explore the potential mechanisms underlying how RIPK3 and RIPK3/FADD pathways prevent excessive accumulation of inflammatory cells, we next evaluated the apoptotic frequency (APF) of various pulmonary cell subsets in *C. neoformans*-infected lungs. Lungs were dissociated gently, and single-cell suspensions were analyzed by flow cytometry with annexin V. Since different leukocyte subsets have distinct life spans and exploit different cell death pathways (30, 31), we first tested their APF in each group without fungal infection to exclude direct effects of FADD and/or RIPK3 deletions. All three groups exhibited similar rates of apoptosis in all subsets, indicating that FADD and RIPK3 were dispensable for homeostatic survival of these immune cells under physiological condition (data not shown).

**Figure 9:**
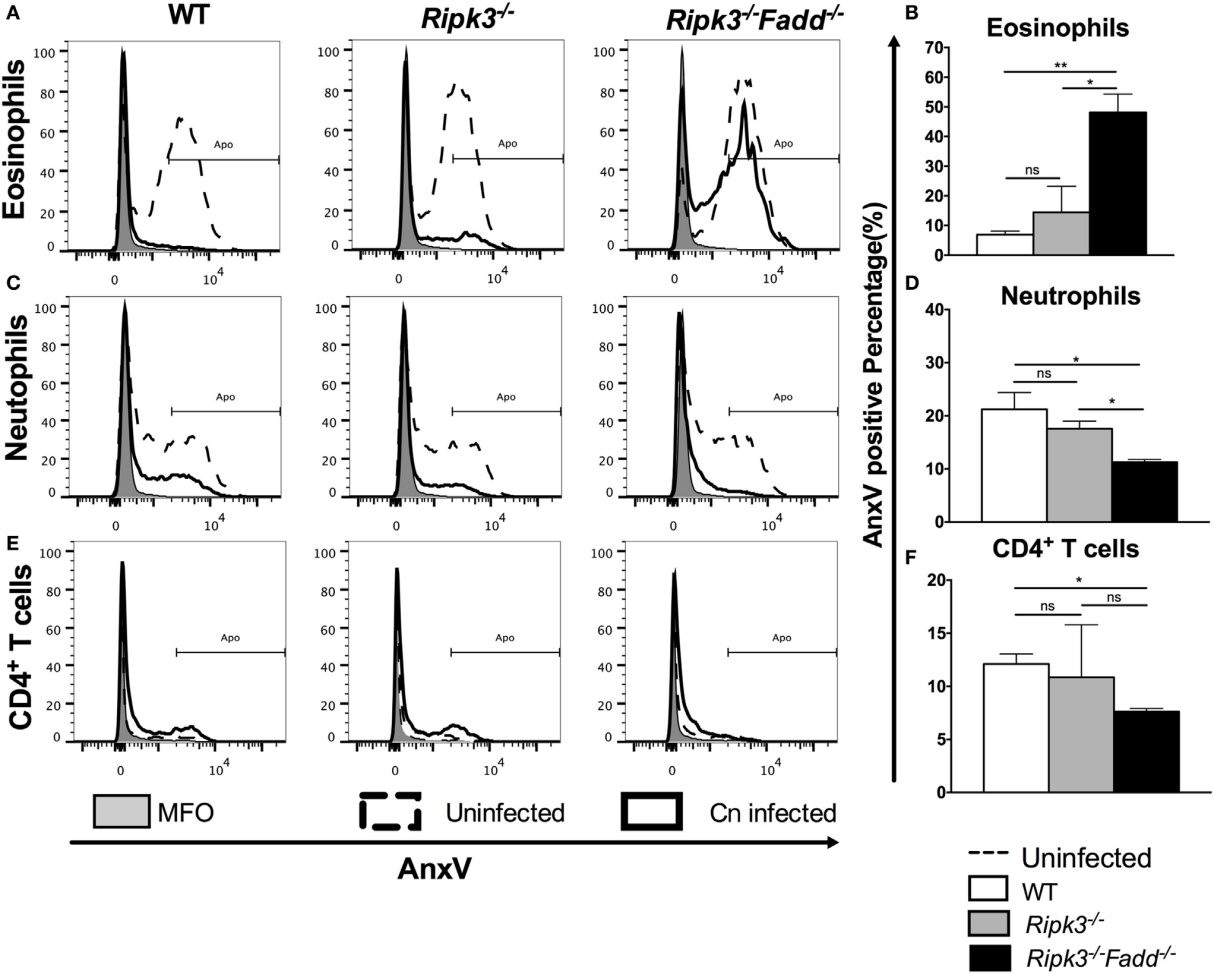
Fas-associated death domain (FADD) deletion differentially regulated the apoptosis of pulmonary leukocyte subsets during *Cryptococcus neoformans* infection. Total pulmonary cells were gently harvested from wild-type (WT), *Ripk3*^−/−^, and *Ripk3*^−/−^*Fadd*^−/−^ mice at 0 or 10 days postinfection. Isolated cells were stained with antibodies specific for annexin V and then analyzed by flow cytometry. After *C. neoformans* infection, eosinophils **(A,B)**, neutrophils **(C,D)**, and CD4^+^ T cells **(E,F)** displayed significant alteration in apoptosis frequency in the lungs from *Ripk3*^−/−^*Fadd*^−/−^ mice but not *Ripk3*^−/−^ mice. Other pulmonary subsets had no significant difference in apoptosis frequency between WT and genetic-defect mice (see Figure S2 in Supplementary Material). ns, no significant difference, *P < 0.05, **P < 0.01, between compared groups. This experiment was repeated twice.

Similar APFs in each cell subset at 10 dpi in *Ripk3*^−/−^ compared with WT mouse infected lungs (Figure 9 and Figure S4 in Supplementary Material) suggested that RIPK3 alone had no effect on inflammatory cell apoptosis during *C. neoformans* infection. However, *C. neoformans* infection significantly reduced eosinophil cell death in WT and *Ripk3*^−/−^ mice (eosinophil APF from uninfected mice: 60.9% ± 1.4% decreasing to 6.6 ± 1.2% and 16.3 ± 8.0, respectively), whereas the absence of FADD completely abolished this phenomenon (Figures 9A,B). A considerable reduction of neutrophil cell death was also detected in each group after fungal infection. Furthermore, consistent with the greatest increase in neutrophil accumulation, the *Ripk3*^−/−^*Fadd*^−/−^ group exhibited significant decrease in the APF of neutrophils (11.0% ± 0.9%) compared with WT (21.5 ± 3.2%, *P* < 0.05) or *Ripk3*^−/−^ mice (17.6 ± 1.5%, Figures 9C,D). Among lymphocyte subsets, only CD4^+^ T cells displayed a significant reduction in cell death frequency in *Ripk3*^−/−^*Fadd*^−/−^ mice (12.1 ± 1.7 vs 7.6 ± 0.5%, *P* < 0.05), while other subpopulations did not differ in each group (Figures 9E,F). Collectively, these data indicated that *C. neoformans* infection affected apoptotic rates of certain leukocyte subsets, but only the joint FADD/RIPK3 deletion led to detectable alterations in the APF in these leukocyte subsets during fungal infection.

### The Effects of RIPK3 and FADD Deletions on Anti-Cryptococcal Defenses Appear to Be Unrelated to Their Role Necroptosis Pathway

**Figure 10:**
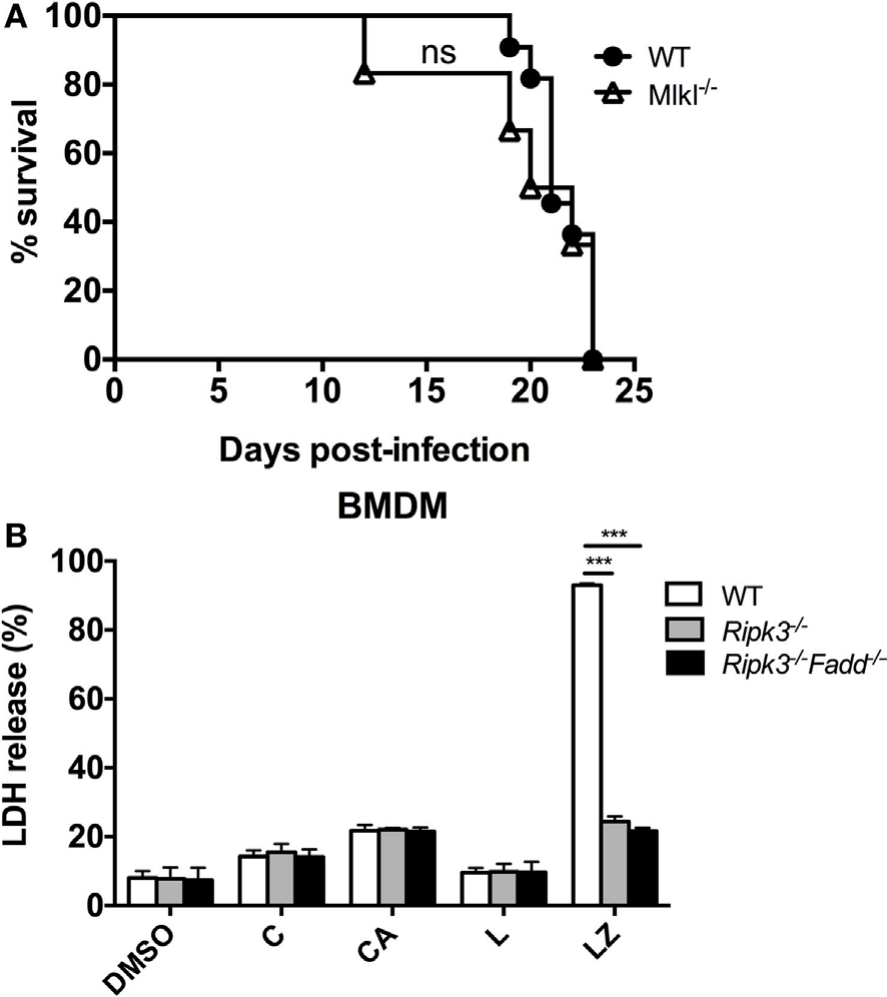
Necroptosis process is not likely a mechanism for RIPK3- and Fas-associated death domain (FADD)-mediated effects on immune responses to *Cryptococcus neoformans*. **(A)** Wild-type (WT) mice and mice with targeted deletion of MLKL gene (distal effector kinase in necroptosis pathway) were infected with *C. neoformans* and analyzed as in Figure 1D. MLKL deletion had no effects on survival, unlike RIPK3 and FADD deletions (per Figure 1D) (*n* = 10 for each group). **(B)** Bone marrow-derived macrophage (BMDM) cells isolated form WT, *Ripk3*^−/−^, *Ripk3*^−/−^*Fadd*^−/−^ mice were challenged with H99 at MOI = 5:1 and co-incubated for 24 h. Supernatants were collected followed by LDH release detection. In RIPK3 or FADD, deletions had no effect on magnitude of BMDM lytic death in contrast with their effects in positive control (LZ). Study was repeated three times independently. Symbols signify C, *C. neoformans*; A, anti-cryptococcal antibody M18B7; Z, zVAD (pan-caspase inhibitor); L, lipopolysaccharide; ns, no significant difference, ***P < 0.001, between compared groups.

RIPK3 and FADD, among other functions, are major intracellular upstream mediators of necroptosis cell death pathway, a PCD resulting in cell lysis, reported to influence mycobacterial-infected macrophages (32) but also to regulate type-I IFN signaling in macrophages independent of necroptosis in influenza-infected macrophages (33). We first asked a question about the global role of necroptosis pathway in anti-cryptococcal host defenses. To asses this, mice with deletion of a distal effector kinase in necroptosis pathway, MLKL along with the WT mice, were infected with *C. neoformans* and mouse survival was monitored. Results show that MLKL deletion had no significant effect on *C. neoformans*-infected mouse survival (Figure 10A) demonstrating that the necroptosis cell death pathway is dispensable for mouse resistance to *C. neoformans*.

We next asked, whether *C. neoformans* infection triggers significant level of lytic cell death in macrophages and whether this was affected by RIPK3 and FADD deletion. To address this question, we used LDH release assay on WT, *Ripk3*^−/−^, *Ripk3*^−/−^*Fadd*^−/−^ BMDM following *in vitro* H99-challenge. Results show that *C. neoformans* infection triggered lytic death in a small subset of BMDM (14.3 ± 1.7%, Figure 10B), The cell death rate was potentiated when the cells were treated with *C. neoformans* opsonized with M18B7 antibody, which increases cryptococcal uptake by macrophages (34) reaching (21.7 ± 1.6%, Figure 10B). However, neither RIPK3 deletion alone nor combined with FADD deletion had effect on the rate of BMDM death. This was in contrast with the positive control, in which lytic cell death triggered by the combined simulation of lipopolysaccharide with pan-caspase inhibition was profoundly reduced *Ripk3*^−/−^, *Ripk3*^−/−^*Fadd*^−/−^ BMDM (Figure 10B). Thus, RIPK3 and FADD are not directly involved in regulation lytic cell death of BMDM population infected with *C. neoformans in vitro*, further supporting that the susceptibility of *Ripk3*^−/−^ mice and *Ripk3*^−/−^*Fadd*^−/−^ mice to cryptococcal infection was mechanistically unrelated to the role RIPK3 and FADD in necroptosis pathway.

## Discussion

While PCD pathway components were shown to be important regulators of the immune responses, the role of FADD and RIPK3 in host defenses against fungal infection remained unknown up to this point. This report provides novel data demonstrating that RIPK3 and FADD are crucial for fungal containment and survival of the infected host during *C. neoformans* infection. Here, we show that these factors serve jointly as physiological “brake” that prevents the development of over exuberant inflammation and profound Th1 bias, which in their absence leads to pulmonary damage and rapid deterioration of the infected host.

Our first group of studies elucidated the involvement of both RIPK3 and FADD during cryptococcal infection. Upon *C. neoformans* infection, RIPK3 expression in infected lungs was strongly upregulated and deletion of RIPK3 significantly shortened the survival of the infected mice and impaired fungal clearance. Furthermore, the absence of RIPK3 resulted in upregulation of pro-inflammatory components including, increased T cell numbers and neutrophils and increased inflammatory cytokines in response to *C. neoformans* (Figures 5 and 6). These data provide evidence that RIPK3 exerts an important role in controlling inflammatory responses with some minor effect on Th polarization during *C. neoformans* infection that is at least in part independent of its well-established function in apoptosis and necroptosis pathways (Figures 9 and 10).

While FADD expression appeared to be constitutive, RIPK3/FADD double deletion resulted in further enhanced susceptibility to *C. neoformans* infection, demonstrating that joint absence of these factors further potentiated the effects of single RIPK3 deletion at both severity of pathology and upregulation of inflammatory components in the infected lungs. In *Ripk3*^−/−^*Fadd*^−/−^ mice, lung pathology and the effect on mouse survival effects were more severe (Figures 1–3; Figure S2 in Supplementary Material) compared to *Ripk3*^−/−^ mice. Our pathology studies further highlighted that in *C. neoformans*-infected lungs RIPK3 and FADD play important roles in protecting infected lungs against the rapid development of severe pathology associated with excessive accumulation and activation of leukocytes. One caveat here could be that the enhanced inflammatory pathology was driven by the increased fungal burden; however, the lung CFU burdens even in the most profoundly affected *Ripk3*^−/−^*Fadd*^−/−^ mice were still relatively modest and could not explain the profound pathological changes and 80% mortality at day 10 in these mice. This, together with very selective amplification of Th1 response, clearly indicates that rather small differences in fungal burdens were not the major driver of the profound changes in the immunophenotype and increase in mouse mortality.

Inflammation is a double-edged sword in the pathogenesis of infectious disease. While suboptimal production of pro-inflammatory cytokines hinders control of *C. neoformans* infection (35–37), highly elevated production of these factors promotes severe inflammatory tissue damage (38). In at least some aspects of host–fungus interactions, a preservation immune strategy denoted “protective tolerance” may be optimal to limit immunopathology while controlling fungal infection (39). This is the first report that RIPK3 and FADD may play jointly important roles by critically fine-tuning “protective tolerance” mechanisms during anti-cryptococcal defense. We show that both of these two molecules are required for optimal control of fungal growth and host protection against the severe inflammatory pathology that develops in lungs in the absence of these factors. Interestingly, the phenotypes that developed in *Ripk3*^−/−^*Fadd*^−/−^ mice mirror many findings in cryptococcosis patients who suffer from IRIS. The development of IRIS (pre-IRIS phase) is specifically characterized by increasing pro-inflammatory responses without efficient clearance of the fungal pathogen (40, 41). Several cohort studies highlighted the relationship of IL-6 signaling and the risk of developing IRIS. For every twofold increase in IL-6 or C-reactive protein, the hazard of IRIS increased by 1.6 and 1.5, respectively (42). Another recent study proposed TNFα, IL-1β, and IL-12 to be predictors of IRIS (43), and these factors found to be elevated as a result of RIPK3 and FADD deletion in *C. neoformans*-infected lungs. Finally, a central IRIS characteristic reproduced in *Ripk3*^−/−^*Fadd*^−/−^ mice is massive systemic elevation of IFNγ, TNFα, and IL-6 (42) (Figure 5).

Besides the findings that mimic cytokine profiles of IRIS patients in our models, we also found the elevated induction of IL-1α and neutrophil recruitment in the lungs of both *Ripk3*^−/−^ and *Ripk3*^−/−^*Fadd*^−/−^ mice. Neutrophil recruitment linked to IL-1α upregulation have been shown to contribute to the development of lethal lung pathology during fungal infection with *Aspergillus* (44). Together, these findings document that responses “designed” to be protective in fungal infections can become highly detrimental to the host, and our data demonstrate such detrimental outcomes following RIPK3 and FADD deletion. Collectively, our study shows that RIPK3 and FADD factors are crucial elements of regulatory network that allows protecting the host from pathological effects of inflammation while supporting clearance of the invasive fungal infection.

Another novel finding was that the expression of RIPK3 and FADD proteins was required for the development of Th2 polarization during *C. neoformans* infection. While the deletion of RIPK3 had subtle effect on Th2 polarization, joint deletion of RIPK3 and FADD resulted in a complete switch from strong Th2 to strong Th1 response. Cytokine profile analysis revealed massive reduction in Th2 cytokines (IL-4 and IL-33) in infected lungs of *Ripk3*^−/−^*Fadd*^−/−^ mice. This, together with diminished GATA3 expression by CD4 T cells and the absence of serum IgE accumulation (Figures 5 and 6), indicates the loss of Th2 polarization in response to RIPK3 and FADD deletion. Th1 and Th2 responses are known to counterbalance each other. Therefore, it remains to be determined if the primary effect of RIPK3/FADD deletion was the lack of the Th2 development with its subsequent “replacement by Th1,” other way around, or else both pathways were subjected to concurrent regulation by joint action of RIPK3 and FADD. While future studies are needed to address these points, our data show that the net effect of RIPK3 and FADD is directly or indirectly counter-regulate Th1 and support Th2 polarization during *C. neoformans* infection. Another highly unexpected finding was that strong Th1 bias and the absence of Th2 rather than being protective (19, 45) resulted in non-protective response in the infected *Ripk3*^−/−^*Fadd*^−/−^ mice. This absence of improved clearance following RIPK3/FADD deletions, despite the robust Th1 polarization, was not due to a defect in macrophage M1 polarization or loss of their intrinsic fungicidal function downstream of robust M1 polarization. Our studies of macrophages from infected lungs and BMDMs revealed neither a defect in macrophage polarization nor a defect in their effector (killing) functions resulting from RIPK3 or FADD deletion (Figure 7). In spite of this, we observed paradoxical impairment of pulmonary fungal clearance in both *Ripk3*^−/−^ and *Ripk3*^−/−^*Fadd*^−/−^ mice (Figure 2). One explanation for this observation is that the excessive systemic activation of microbicidal factors in macrophages resulted in exhaustion of these effectors cells even before they reached the infection site, which seems to be consistent with our data (Figures 4 and 7). Additionally, the excessive accumulation of neutrophils at the infection site interfered with macrophage and T-cell fungicidal functions, since an excessive accumulation of neutrophils have been reported to contribute to tissue damage and defects in the clearance of other fungal organisms (46, 47).

The final observation in our study is quite strong independence of immunoregulatory effects exerted by RIPK3 and FADD from apoptotic or necroptotic cell death. The diminished apoptosis likely contributed to the excessive accumulation of neutrophils in infected *Ripk3*^−/−^*Fadd*^−/−^ mice (Figures 3, 4B and 9D) since, granulocytes are typically eliminated *via* extrinsic apoptosis after a brief period of activation (48). However, the enhanced neutrophil accumulation was also observed in *Ripk3*^−/−^ mice without significant effect on neutrophil APF. Likewise, APF in eosinophil population was greatest in the infected *Ripk3*^−/−^*Fadd*^−/−^ mice, demonstrating that eosinophil apoptosis did not require or was positively regulated by FADD or RIPK3. Furthermore, RIPK3 and FADD deletion appeared not to affect APF in T and B cells, monocytes, DCs, or epithelial cells in the infected lungs. Finally, our outcomes do not favor necroptosis as an important pathway defining host–pathogen interactions during *C. neoformans* infection (Figure 10). Thus, while future studies are needed to provide definitive answers, our data favor the hypothesis that RIPK3 and FADD can induce immunoregulatory effects during fungal infection in a manner independent of their role in PCD responses, but chiefly by regulating cytokine responses.

In summary, our results demonstrate, for the first time, that RIPK3 and FADD are vital components of the immune responses to fungal pathogens. These molecules are required for fine-tuning of inflammatory responses during infection, acting as powerful regulators of Th1 and Th2 polarization. They contribute to optimal fungal clearance and serve as indispensable “nodes” in immunoregulatory network supporting tissue damage control in fungal-infected host.

## Ethics Statement

This study was carried out in accordance with the recommendations of National Institutes of Health Guide for the Care and Use of Laboratory Animals. The protocol was approved by the Scientific Investigation Board of Second Military Medical University.

## Author Contributions

ZF, QX, and WF contributed to study concept and design, performing the experiments, and drafting of the manuscript. WL, MO, XD, and HZ (Haibing Zhang) contributed to study concept and design, analysis, and interpretation of data, critical revision of the manuscript, obtained funding, and provided study supervision, administrative, and technical support. HZ (Haiwei Zhang), JX (Jintao Xu), WP, and JX (Jinhua Xu) contributed to data analysis and revision of the manuscript. All listed authors gave final approval of the manuscript.

## Conflict of Interest Statement

The authors declare that the research was conducted in the absence of any commercial or financial relationships that could be construed as a potential conflict of interest.

## References

[1] Fang W Fa Z Liao W Epidemiology of *Cryptococcus* and cryptococcosis in China. Fungal Genet Biol (2015) 78:7–15. 10.1016/j.fgb.2014.10.017 25445309

[2] Heitman J Kozel TR Kwon-Chung KJ Perfect JR Casadevall A Cryptococcus: From Human Pathogen to Model Yeast. Washington, DC: ASM Press (2010). 275 p.

[3] Maziarz EK Perfect JR. Cryptococcosis. Infect Dis Clin North Am (2016) 30(1):179–206. 10.1016/j.idc.2015.10.006 26897067 PMC5808417

[4] Greenlee-Wacker MC. Clearance of apoptotic neutrophils and resolution of inflammation. Immunol Rev (2016) 273(1):357–70. 10.1111/imr.12453 27558346 PMC5000862

[5] Ortega-Gomez A Perretti M Soehnlein O. Resolution of inflammation: an integrated view. EMBO Mol Med (2013) 5(5):661–74. 10.1002/emmm.201202382 23592557 PMC3662311

[6] Scaffidi C Volkland J Blomberg I Hoffmann I Krammer PH Peter ME. Phosphorylation of FADD/MORT1 at serine 194 and association with a 70-kDa cell cycle-regulated protein kinase. J Immunol (2000) 164(3):1236–42. 10.4049/jimmunol.164.3.1236 10640736

[7] Zhang J Kabra NH Cado D Kang C Winoto A. FADD-deficient T cells exhibit a disaccord in regulation of the cell cycle machinery. J Biol Chem (2001) 276(32):29815–8. 10.1074/jbc.M103838200 11390402

[8] Balachandran S Thomas E Barber GN. A FADD-dependent innate immune mechanism in mammalian cells. Nature (2004) 432(7015):401–5. 10.1038/nature03124 15549108

[9] Ma Y Liu H Tu-Rapp H Thiesen HJ Ibrahim SM Cole SM Fas ligation on macrophages enhances IL-1R1-toll-like receptor 4 signaling and promotes chronic inflammation. Nat Immunol (2004) 5(4):380–7. 10.1038/ni1054 15004557

[10] Zhang J Cado D Chen A Kabra NH Winoto A. Fas-mediated apoptosis and activation-induced T-cell proliferation are defective in mice lacking FADD/Mort1. Nature (1998) 392(6673):296–300. 10.1038/32681 9521326

[11] Varfolomeev EE Schuchmann M Luria V Chiannilkulchai N Beckmann JS Mett IL Targeted disruption of the mouse caspase 8 gene ablates cell death induction by the TNF receptors, Fas/Apo1, and DR3 and is lethal prenatally. Immunity (1998) 9(2):267–76. 10.1016/S1074-7613(00)80609-3 9729047

[12] He S Wang L Miao L Wang T Du F Zhao L Receptor interacting protein kinase-3 determines cellular necrotic response to TNF-alpha. Cell (2009) 137(6):1100–11. 10.1016/j.cell.2009.05.021 19524512

[13] Kasof GM Prosser JC Liu D Lorenzi MV Gomes BC. The RIP-like kinase, RIP3, induces apoptosis and NF-kappaB nuclear translocation and localizes to mitochondria. FEBS Lett (2000) 473(3):285–91. 10.1016/S0014-5793(00)01473-3 10818227

[14] Moriwaki K Balaji S McQuade T Malhotra N Kang J Chan FK. The necroptosis adaptor RIPK3 promotes injury-induced cytokine expression and tissue repair. Immunity (2014) 41(4):567–78. 10.1016/j.immuni.2014.09.016 25367573 PMC4220270

[15] Shlomovitz I Zargrian S Gerlic M. Mechanisms of RIPK3-induced inflammation. Immunol Cell Biol (2017) 95(2):166–72. 10.1038/icb.2016.124 27974745

[16] Zhang X Fan C Zhang H Zhao Q Liu Y Xu C MLKL and FADD are critical for suppressing progressive lymphoproliferative disease and activating the NLRP3 inflammasome. Cell Rep (2016) 16(12):3247–59. 10.1016/j.celrep.2016.06.103 27498868 PMC7191534

[17] Semighini CP Averette AF Perfect JR Heitman J. Deletion of *Cryptococcus neoformans* AIF ortholog promotes chromosome aneuploidy and fluconazole-resistance in a metacaspase-independent manner. PLoS Pathog (2011) 7(11):e1002364. 10.1371/journal.ppat.1002364 22114551 PMC3219705

[18] Meng Y Zhang C Yi J Zhou Z Fa Z Zhao J Deubiquitinase Ubp5 is required for the growth and pathogenicity of *Cryptococcus gattii*. PLoS One (2016) 11(4):e0153219. 10.1371/journal.pone.0153219 27049762 PMC4822882

[19] Zhang Y Wang F Tompkins KC McNamara A Jain AV Moore BB Robust Th1 and Th17 immunity supports pulmonary clearance but cannot prevent systemic dissemination of highly virulent *Cryptococcus neoformans* H99. Am J Pathol (2009) 175(6):2489–500. 10.2353/ajpath.2009.090530 19893050 PMC2789623

[20] Qiu Y Dayrit JK Davis MJ Carolan JF Osterholzer JJ Curtis JL Scavenger receptor A modulates the immune response to pulmonary *Cryptococcus neoformans* infection. J Immunol (2013) 191(1):238–48. 10.4049/jimmunol.1203435 23733871 PMC4007509

[21] Stevens WW Kim TS Pujanauski LM Hao X Braciale TJ. Detection and quantitation of eosinophils in the murine respiratory tract by flow cytometry. J Immunol Methods (2007) 327(1–2):63–74. 10.1016/j.jim.2007.07.011 17716680 PMC2670191

[22] Xu J Eastman AJ Flaczyk A Neal LM Zhao G Carolan J Disruption of early tumor necrosis factor alpha signaling prevents classical activation of dendritic cells in lung-associated lymph nodes and development of protective immunity against cryptococcal infection. MBio (2016) 7(4):e00510–16. 10.1128/mBio.00510-16 27406560 PMC4958242

[23] Szarka RJ Wang N Gordon L Nation PN Smith RH. A murine model of pulmonary damage induced by lipopolysaccharide via intranasal instillation. J Immunol Methods (1997) 202(1):49–57. 10.1016/S0022-1759(96)00236-0 9075771

[24] Davis MJ Tsang TM Qiu Y Dayrit JK Freij JB Huffnagle GB Macrophage M1/M2 polarization dynamically adapts to changes in cytokine microenvironments in *Cryptococcus neoformans* infection. MBio (2013) 4(3):e00264–13. 10.1128/mBio.00264-13 23781069 PMC3684832

[25] Davis MJ Eastman AJ Qiu Y Gregorka B Kozel TR Osterholzer JJ *Cryptococcus neoformans*-induced macrophage lysosome damage crucially contributes to fungal virulence. J Immunol (2015) 194(5):2219–31. 10.4049/jimmunol.1402376 25637026 PMC4379045

[26] Alanio A Desnos-Ollivier M Dromer F Dynamics of *Cryptococcus neoformans*-macrophage interactions reveal that fungal background influences outcome during cryptococcal meningoencephalitis in humans. MBio (2011) 2(4):e00158–11. 10.1128/mBio.00158-11 21828220 PMC3149853

[27] Arora S Olszewski MA Tsang TM McDonald RA Toews GB Huffnagle GB. Effect of cytokine interplay on macrophage polarization during chronic pulmonary infection with *Cryptococcus neoformans*. Infect Immun (2011) 79(5):1915–26. 10.1128/IAI.01270-10 21383052 PMC3088136

[28] Herring AC Falkowski NR Chen GH McDonald RA Toews GB Huffnagle GB Transient neutralization of tumor necrosis factor alpha can produce a chronic fungal infection in an immunocompetent host: potential role of immature dendritic cells. Infect Immun (2005) 73(1):39–49. 10.1128/IAI.73.1.39-49.2005 15618139 PMC538928

[29] Eastman AJ Osterholzer JJ Olszewski MA. Role of dendritic cell-pathogen interactions in the immune response to pulmonary cryptococcal infection. Future Microbiol (2015) 10(11):1837–57. 10.2217/fmb.15.92 26597428 PMC5720351

[30] Hampson P Hazeldine J Lord JM. Neutrophil apoptosis and its induction as a potential treatment for chronic inflammatory disease. Curr Opin Hematol (2013) 20(1):10–5. 10.1097/MOH.0b013e32835b06be 23196895

[31] Shen ZJ Malter JS. Determinants of eosinophil survival and apoptotic cell death. Apoptosis (2015) 20(2):224–34. 10.1007/s10495-014-1072-2 25563855 PMC5798882

[32] Zhao X Khan N Gan H Tzelepis F Nishimura T Park SY Bcl-xL mediates RIPK3-dependent necrosis in *M. tuberculosis*-infected macrophages. Mucosal Immunol (2017). 10.1038/mi.2017.12 28401933 PMC5638669

[33] Downey J Pernet E Coulombe F Allard B Meunier I Jaworska J RIPK3 interacts with MAVS to regulate type I IFN-mediated immunity to influenza A virus infection. PLoS Pathog (2017) 13(4):e1006326. 10.1371/journal.ppat.1006326 28410401 PMC5406035

[34] Zebedee SL Koduri RK Mukherjee J Mukherjee S Lee S Sauer DF Mouse-human immunoglobulin G1 chimeric antibodies with activities against *Cryptococcus neoformans*. Antimicrob Agents Chemother (1994) 38(7):1507–14. 10.1128/AAC.38.7.1507 7979280 PMC284584

[35] Bauman SK Huffnagle GB Murphy JW. Effects of tumor necrosis factor alpha on dendritic cell accumulation in lymph nodes draining the immunization site and the impact on the anticryptococcal cell-mediated immune response. Infect Immun (2003) 71(1):68–74. 10.1128/IAI.71.1.68-74.2003 12496150 PMC143367

[36] Siddiqui AA Shattock RJ Harrison TS. Role of capsule and interleukin-6 in long-term immune control of *Cryptococcus neoformans* infection by specifically activated human peripheral blood mononuclear cells. Infect Immun (2006) 74(9):5302–10. 10.1128/IAI.00661-06 16926424 PMC1594853

[37] Axton PJ Bancroft GJ In vivo analysis of immune responses to *Cryptococcus neoformans* – role of interferon-gamma in host resistance. Biochem Soc Trans (1997) 25(2):276S 10.1042/bst025276s 9191320

[38] Osterholzer JJ Milam JE Chen GH Toews GB Huffnagle GB Olszewski MA. Role of dendritic cells and alveolar macrophages in regulating early host defense against pulmonary infection with *Cryptococcus neoformans*. Infect Immun (2009) 77(9):3749–58. 10.1128/IAI.00454-09 19564388 PMC2737986

[39] Roussey JA Olszewski MA Osterholzer JJ Immunoregulation in fungal diseases. Microorganisms (2016) 4(4):E47 10.3390/microorganisms4040047 27973396 PMC5192530

[40] Skiest DJ Hester LJ Hardy RD. Cryptococcal immune reconstitution inflammatory syndrome: report of four cases in three patients and review of the literature. J Infect (2005) 51(5):e289–97. 10.1016/j.jinf.2005.02.031 16321643

[41] Wiesner DL Boulware DR. *Cryptococcus*-related immune reconstitution inflammatory syndrome (IRIS): pathogenesis and its clinical implications. Curr Fungal Infect Rep (2011) 5(4):252–61. 10.1007/s12281-011-0064-8 22389746 PMC3289516

[42] Boulware DR Meya DB Bergemann TL Wiesner DL Rhein J Musubire A Clinical features and serum biomarkers in HIV immune reconstitution inflammatory syndrome after cryptococcal meningitis: a prospective cohort study. PLoS Med (2010) 7(12):e1000384. 10.1371/journal.pmed.1000384 21253011 PMC3014618

[43] Sun J Chen H Xie Y Su J Huang Y Xu L Nuclear factor of activated T cells and cytokines gene expression of the T cells in AIDS patients with immune reconstitution inflammatory syndrome during highly active antiretroviral therapy. Mediators Inflamm (2017) 2017:1754741. 10.1155/2017/1754741 28316373 PMC5337872

[44] Caffrey AK Lehmann MM Zickovich JM Espinosa V Shepardson KM Watschke CP IL-1alpha signaling is critical for leukocyte recruitment after pulmonary *Aspergillus fumigatus* challenge. PLoS Pathog (2015) 11(1):e1004625 10.1371/journal.ppat.1004625 25629406 PMC4309569

[45] Holmer SM Evans KS Asfaw YG Saini D Schell WA Ledford JG Impact of surfactant protein D, interleukin-5, and eosinophilia on cryptococcosis. Infect Immun (2014) 82(2):683–93. 10.1128/IAI.00855-13 24478083 PMC3911392

[46] Zelante T De Luca A D’Angelo C Moretti S Romani L. IL-17/Th17 in anti-fungal immunity: what’s new? Eur J Immunol (2009) 39(3):645–8. 10.1002/eji.200839102 19283705

[47] Zelante T De Luca A Bonifazi P Montagnoli C Bozza S Moretti S IL-23 and the Th17 pathway promote inflammation and impair antifungal immune resistance. Eur J Immunol (2007) 37(10):2695–706. 10.1002/eji.200737409 17899546

[48] Fox S Leitch AE Duffin R Haslett C Rossi AG. Neutrophil apoptosis: relevance to the innate immune response and inflammatory disease. J Innate Immun (2010) 2(3):216–27. 10.1159/000284367 20375550 PMC2956014

