# Supplementary material for: RIPK3/Fas-Associated Death Domain Axis Regulates Pulmonary Immunopathology to Cryptococcal Infection Independent of Necroptosis
Source: Front Immunol. 2017 Sep 1;8:1055. doi: 10.3389/fimmu.2017.01055 (PMC5585137; doi:10.3389/fimmu.2017.01055)
Supplement: Supplementary file 1 [file Presentation_1.PDF]

Supplemental Information

Table S1. Primers for real-time PCR in this study.

Primer name	Primer sequence (5’->3’)
TNFα-F	CCTGTAGCCCACGTCGTAGC
TNFα-R	AGCAATGACTCCAAAGTAGACC
IFNγ-F	CAAAAGGATGGTGACATGAA
IFNγ-R	TTGGCAATACTCATGAATGC
IL-4F	ACGAGGTCACAGGAGAAGGGA
IL-4R	TTGGAAGCCCTACAGACGAGC
IL-5F	GCAATGGAAGGCTGAGGCTG
IL-5R	GGGTATGTGATCCTCCTGCGTC
IL-6F	GACAAAGCCAGAGTCCTTCAGAGAG
IL-6R	CTAGGTTTGCCGAGTAGATCT
IL-10F	CTATGCTGCCTGCTCTTACTG
IL-10R	CATGGCCTTGTAGACACCTTG
IL-12F	TCAATCACGCTACCTCCTC
IL-12R	CTTGTTGTGGAAGAAGTCTCTC
IL-33F	GATGGGAAGAAGGTGATGGTG

IL-33R	TTGTGAAGGACGAAGAAGGC
KC-F	ACTGGGATTCACCTCAAGAA
KC-R	TCTCCGTTACTTGGGGACAC
Arg1-F	CAGAAGAATGGAAGAGTCAG
Arg1-R	CAGATATGCAGGGAGTCACC
Fizz-F	TTCTTGCCAATCCAGCTAAC
Fizz-R	GGGTTCTCCACCTCTTCATT
iNOS-F	TTTGCTTCCATGCTAATGCGAAAG
iNOS-R	GCTCTGTTGAGGTCTAAAGGCTCCG
GAPDH-F	AGGTCGGTGAACGGATTTG
GAPDH-R	TGTAGACCATGTAGTTGAGGTCA

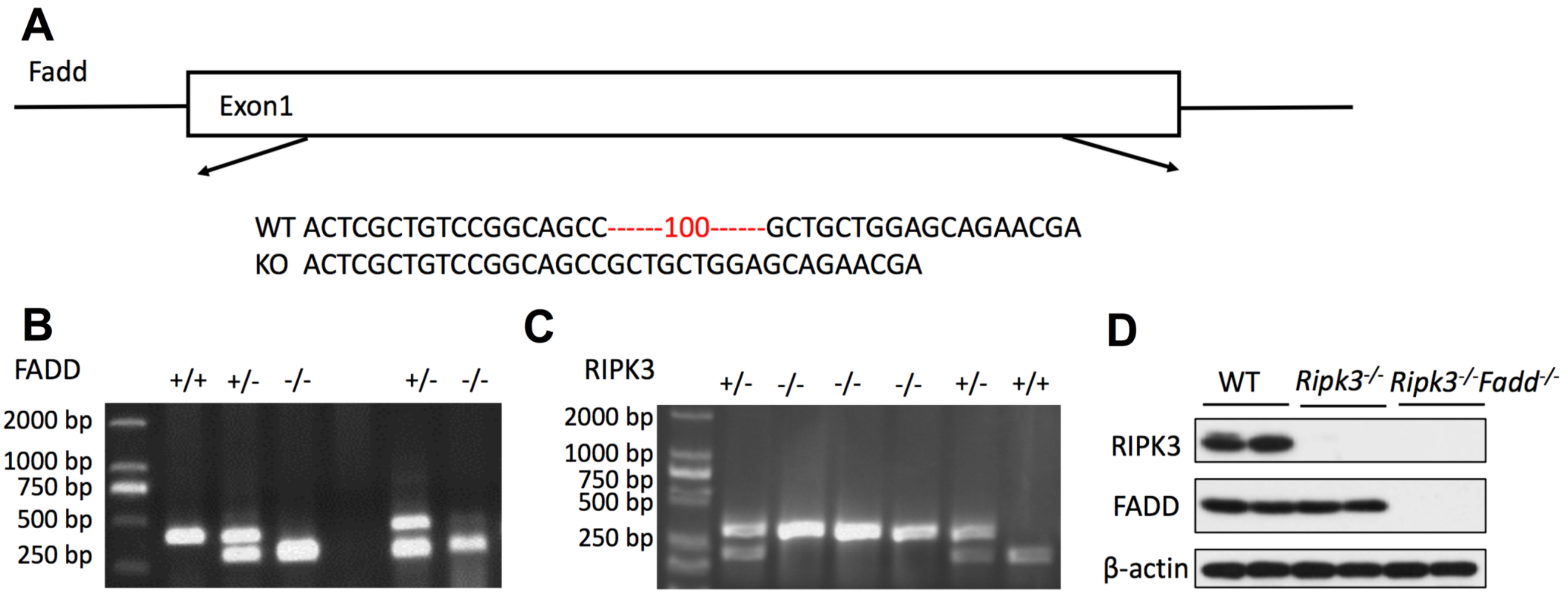

FigureS1 Generation of *Ripk3$^{-/-}$Fadd$^{-/-}$* and *Ripk3$^{-/-}$* Mice. (A) A 100-bp deletion was introduced into exon 1 of the Fadd gene. *Ripk3$^{-/-}$* mice were crossed with *Fadd$^{+/-}$* mice to obtain *Ripk3$^{-/-}$Fadd$^{-/-}$* mice. (B) Mice of WT (+/+), Heterozygous (+/-) and Fadd knockout (-/-) were genotyped by PCR (C) Mice of WT (+/+), Heterozygous (+/-) and Ripk3 knockout (-/-) were genotyped by PCR. (D) RIPK3 and FADD expression in lung tissues from WT, *Ripk3$^{-/-}$*, *Ripk3$^{-/-}$Fadd$^{-/-}$* mice respectively.

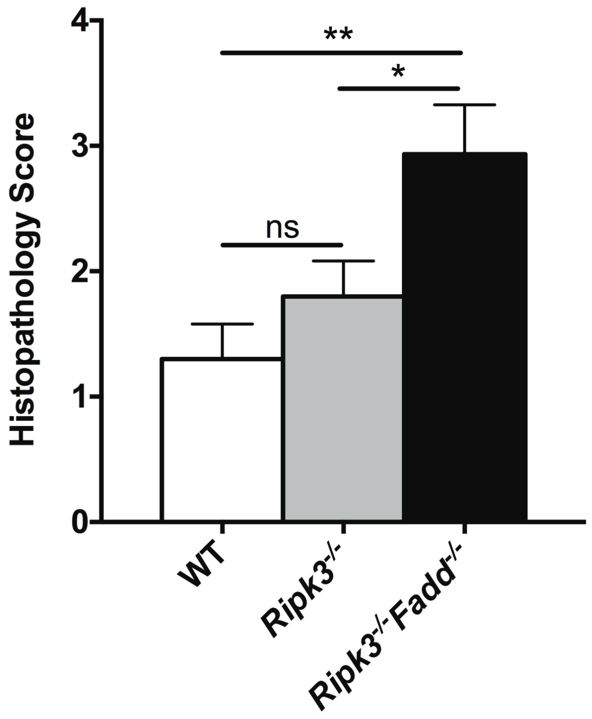

FigureS2. Deletion of RIPK3 and FADD resulted in enhanced inflammatory infiltration and tissue injury following pulmonary infection with *C. neoformans*. Lung tissue inflammation and injury score were performed in blinded fashion by 3 different pathology lab technicians and averaged. ns: no significant difference, * $P<0.05$, ** $P<0.01$, between compared groups.

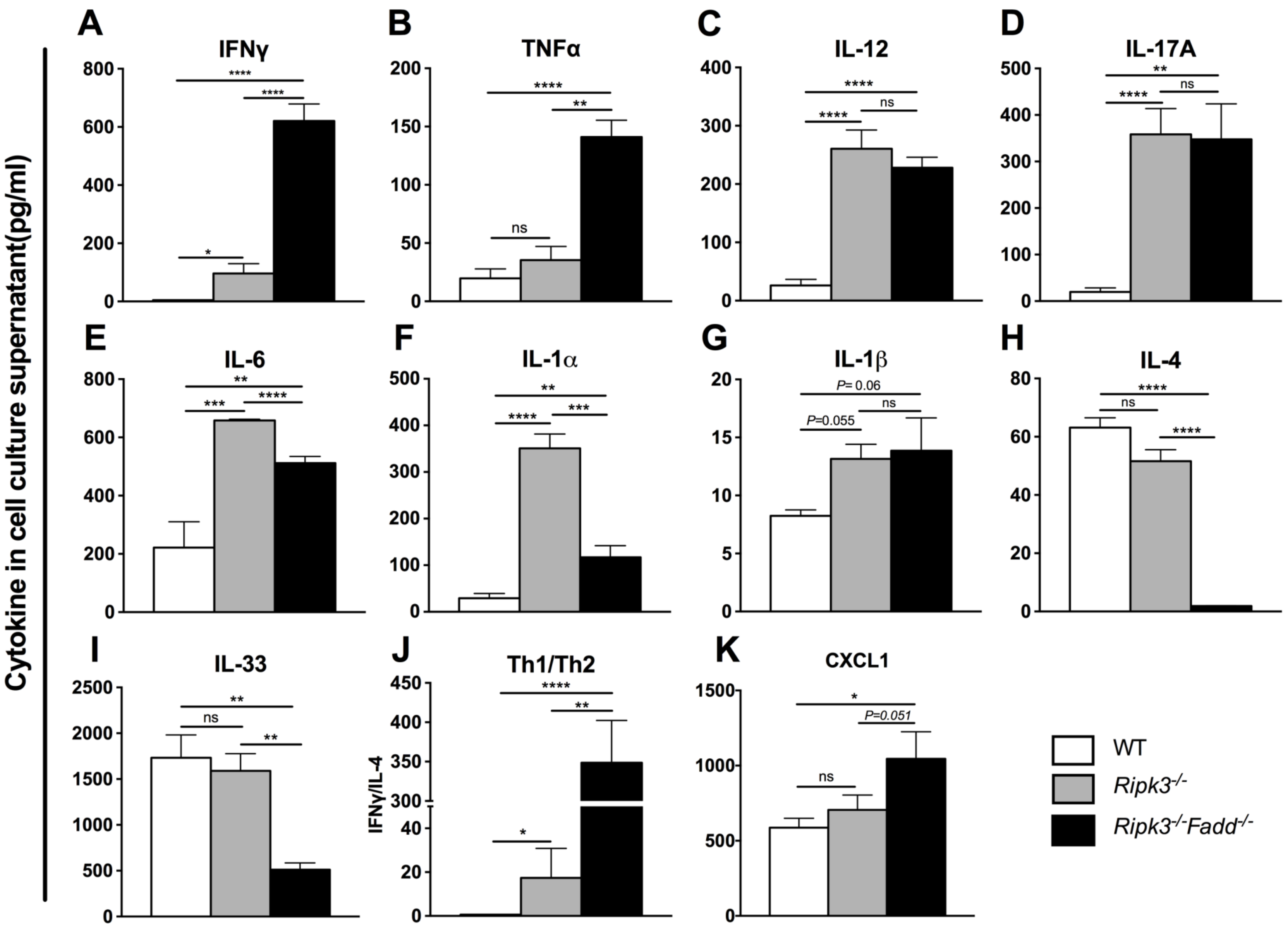
Cytokine in cell culture supernatant(pg/ml)
A
IFNγ
B
TNFα
C
IL-12
D
IL-17A
E
IL-6
F
IL-1α
G
IL-1β
P= 0.06
P=0.055
H
IL-4
I
IL-33
J
Th1/Th2
IFNγ/IL-4
K
CXCL1
P=0.051
WT
Ripk3-/-
Ripk3-/-Fadd-/-

FigureS3. RIPK3 and RIPK3/FADD deletions altered cytokine profiles secreted by cultured leukocytes isolated from *C. neoformans*-infected lungs. Lung leukocytes were harvested from WT, *Ripk3*$^{-/-}$, or *Ripk3*$^{-/-}$*Fadd*$^{-/-}$mice at 10 dpi with *C. neoformans* infection. Isolated leukocytes were cultured for 24 hours at 5×10^{6} cells/ml in the absence of any stimulation. Cytokine levels were detected in the supernatants of cell cultures by ELISA. Values represent mean cytokine concentrations (**A-I** and **K**, pg/ml) or **(J)**, the IFN-γ/IL-4 ratio. *n*= 6 mice per group. ns: no significant difference, * *P*<0.05, ** *P*<0.01, *** *P*<0.001, **** *P*<0.0001, between compared groups. This experiment was repeated twice.

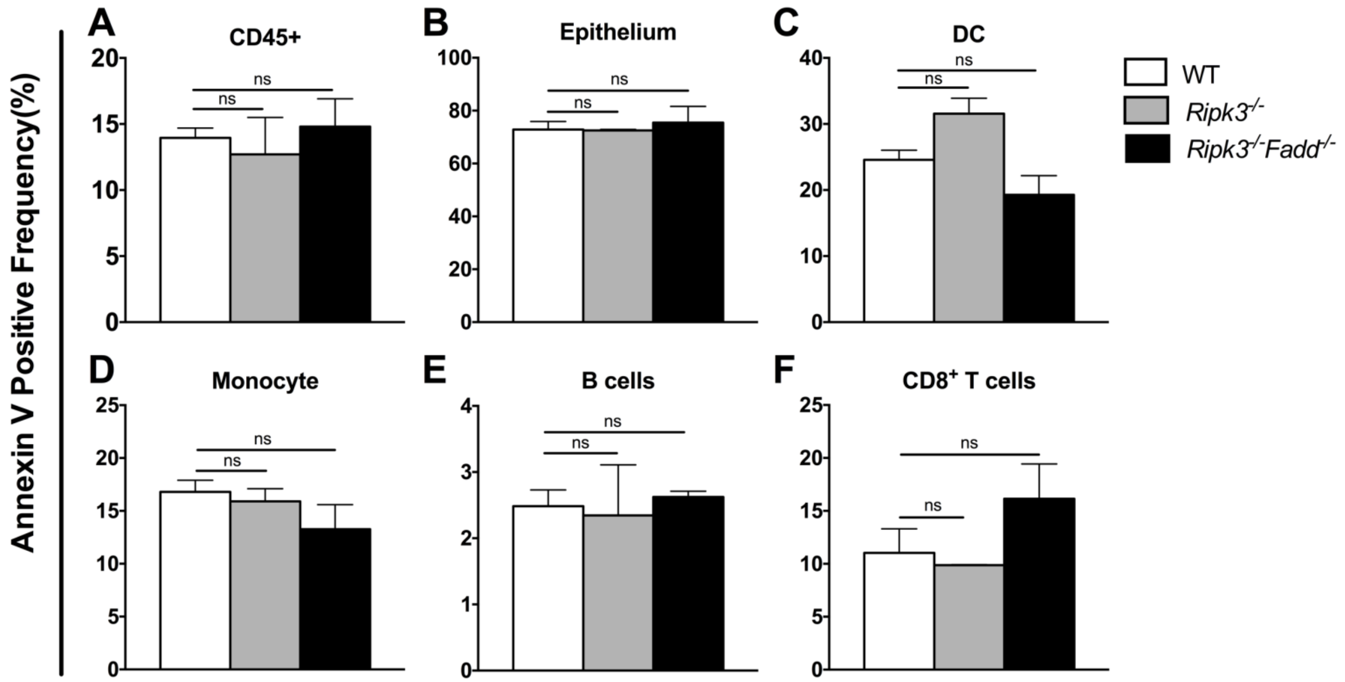

Figure S4. FADD deletion differentially regulated the apoptosis of pulmonary subsets during *C. neoformans* infection. Except eosinophils, neutrophils, and $CD4^+$ T cells (Figure 8), other subsets had no significant difference in apoptosis frequency between WT and genetic-defect mice. ns: no significant difference.
